# Surfactant-Nanoparticle Formulations for Enhanced Oil Recovery in Calcite-Rich Rocks

**DOI:** 10.1021/acs.langmuir.4c03100

**Published:** 2024-11-12

**Authors:** Hosein Rezvani, Bernard P. Binks, Duy Nguyen

**Affiliations:** † Department of Chemistry, University of Hull, Hull HU6 7RX, U.K.; ‡ ChampionX, 11177 S. Stadium Drive, Sugar Land, Texas 77478, United States

## Abstract

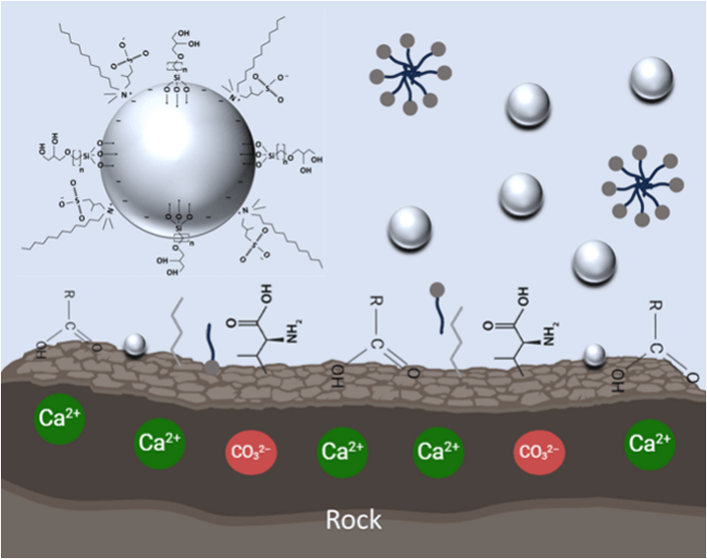

Aqueous surfactant-nanoparticle mixtures have received great attention recently for promoting a more sustainable and efficient enhanced oil recovery (EOR) process. However, colloidal stability under reservoir conditions is considered a great challenge. In addition, the way synergy operates in EOR is not clearly understood. This study aims to formulate a cost-effective surfactant-nanoparticle mixture in a formation brine for efficient EOR in calcite-rich oil reservoirs. For this, bare silica nanoparticles were covalently grafted using an epoxysilane and blended with two commercial surfactants, namely a zwitterionic alkyl hydroxysultaine (AHS) and a binary zwitterionic-nonionic (ZN) surfactant, for both additional steric stabilization and EOR. The effects of additives alone or their mixtures were examined at solid–fluid and fluid–fluid interfaces to explore their impact on EOR. The surfactant-nanoparticle blend often showed a pH-responsive behavior at solid–fluid and fluid–fluid interfaces with particles serving as carriers or surface activity improvers for surfactant resulting in different extents of rock wettability alteration and emulsification. In oil recovery tests, optimum surfactant concentrations were found to significantly increase crude oil recovery of formation brine by 36 ± 1% original oil in place (OOIP) in secondary spontaneous imbibition which was further enhanced by 14 ± 0.5% OOIP upon adding a low particle concentration (0.01 wt %). The surfactant-nanoparticle formulation was also efficient in producing residual crude oil in tertiary mode (6% OOIP additional oil recovery after formation brine). The oil recovery results disclosed a high dependence on the emulsification ability of the blends with AHS-particle dispersions producing more stable emulsions and thus more crude oil compared to that of ZN.

## 1 Introduction

Metal oxide particles are versatile and have remarkable applications at fluid–fluid or fluid–solid interfaces. Their nanoforms (<100 nm) have attracted substantial interest in enhanced oil recovery (EOR) applications. Yet, the primary obstacle to overcome pertains to ensuring their long-term colloidal stability under elevated temperatures and high salinity conditions.^1^ The tiny size of nanoparticles enables them to enter deep into the reservoir improving oil displacement. The available literature includes a range of EOR mechanisms associated with nanoparticles. They can alter rock wettability through both adsorption on rock surfaces and the imposition of disjoining pressure leading to increased oil production.^2^ Additionally, they can also form Pickering emulsions to enhance oil movement. Their efficiency extends to reducing oil viscosity and increasing water viscosity (if at high concentration) to lower the mobility ratio and improve oil displacement.^3^ Certain nanoparticles demonstrate the capability to mitigate asphaltene precipitation.^4^ Moreover, they can temporarily obstruct pore channels, raise pressure drops and divert injected fluid toward unswept areas boosting oil displacement.^5^

On the other hand, surfactant flooding has been proven effective for EOR in conventional and more recently unconventional oil reservoirs. The main idea is that amphiphilic surfactants can reduce the oil–water interfacial tension, emulsify oil and water, lower rock hydrophobicity and prevent asphaltene precipitation.^6^ However, hydrolysis, degradation, precipitation and high adsorption onto rock surfaces are prevalent issues with surfactants rendering them expensive and less efficient in some applications.^7^ Several additives have been explored to address surfactant adsorption on rocks with a specific emphasis on metal oxide nanoparticles. These materials are preferred for their environmental friendliness and cost-effectiveness compared to polymers and alkalis.

The existing literature provides evidence of synergistic effects in EOR through the combination of surfactants and nanoparticles.^8^ Beyond the previously covered effects of each chemical, their combination improves their efficacy in EOR by altering surface properties, modifying adsorption behavior and colloidal stabilizing effects. For example, oppositely charged surfactants may adsorb on nanoparticles rendering them hydrophobic enabling their adsorption at the oil–water interface leading to emulsification without the need for an ultralow oil–water interfacial tension.^9^ Or like-charged nanoparticles can further increase surfactant surface activity at oil–water and rock-water interfaces resulting in increased oil recovery.^10^ The suggested mixtures may also result in lower operating costs because of their lower overall chemical concentrations.^11−15^

Zhao et al. recorded an imbibition recovery of 30% original oil in place (OOIP) using a blend of sulfonated silicon quantum dots and bitetradecyl sulfobetaine compared to a particle dispersion alone (13% OOIP) or surfactant solution alone (17% OOIP) which was linked to the oil–water interfacial tension reduction and rock wettability alteration by the blend.^16^ Al-Shatty et al. reported that the blend of hydrophobic octanoic acid-coated alumina nanoparticles and cetyltrimethylammonium bromide (CTAB) increases the oil recovery by 5% OOIP compared to CTAB injection alone due to the higher affinity of particle-surfactant assemblies for the oil–water interface.^17^ de O. Pereira et al. observed an additional oil recovery of 30% OOIP by the blend of Fe_3_O_4_ nanoparticles and CTAB in tertiary mode after secondary brine injection which was related to the easier oil displacement by nanoparticles (through asphaltene adsorption and rock wettability alteration) and surfactant (through oil–water interfacial tension decrease).^18^ Using a mixture of silicon quantum dots and 0.1 wt % alkyl betaine surfactant in 15 wt % synthetic brine, Zhou et al. demonstrated that compared to surfactant alone the particle-surfactant mixture could recover an additional 7.5 and 12% OOIP in spontaneous imbibition and flooding respectively, in Bakken cores. Apart from oil–water interfacial tension reduction, the self-layering of particles within the wedge layer confined between the oil droplet and the rock surface was identified as the factor responsible for generating disjoining pressure ultimately enhancing the movement of oil droplets.^19^ Pang and Mohanty studied different nanoparticle-surfactant mixtures in high salinity brine for both EOR and CO_2_ storage. They observed that particle addition can significantly enhance foam half-life (120 to 540 min) and apparent viscosity (29 to 43 cP) at reservoir conditions resulting in larger oil production and CO_2_ storage.^15^

As reviewed, aqueous particle-surfactant mixtures are promising for EOR. However, challenges arise in determining the appropriate nanoparticle and surfactant type, ensuring nanoparticle stabilization at reservoir conditions and chemical concentrations.^20^ Compared to conventional surfactants, zwitterionic and nonionic surfactants have gained great attention for EOR due to their perfect characteristics (Table S1). Elevated temperature and salinity can cause nanoparticles to aggregate and change the adsorption behavior of both particles and surfactants at fluid–fluid and fluid–solid interface lowering their efficacy.^11,15^

In this study, commercial zwitterionic and binary zwitterionic-nonionic (ZN) surfactants were used. These surfactants have undergone prior screening and evaluation and have been identified as potential candidates for EOR by the sponsor (ChampionX). The ZN surfactant benefited from a proprietary demulsifier to prevent stable emulsions in surface facilities—a critical component for field applications. The objective of this study was to identify effective formulations of nanoparticle-surfactant mixtures for EOR in calcite-rich rocks. Initially, it focused on achieving long-term stabilization of silica particles in a formation brine at high temperatures (75 °C). Subsequently, the study investigated the impact of nanoparticles, surfactants and their blends on reservoir properties such as rock wettability, oil–water interfacial tension and oil–water emulsification. Additionally, it analyzed surfactant adsorption onto rock surfaces and examined how particle addition affects this adsorption. The study emphasized using low particle concentrations to minimize formation damage and improve the cost-effectiveness of field applications, contrasting with high concentrations typically used in similar research.^21^

## 2 Experimental Section

### 2.1 Materials

Elga reverse osmosis and Milli-Q ultrafiltration systems (Millipore, France) were used to purify water (pH = 6.5, ρ = 0.998 g cm^–3^, air–water surface tension at 25 °C σ_aw_ = 72 ± 0.1 mN m^–1^). The oilfield formation brine, Permian brine, had a total salinity of 12.6 wt %. It was prepared by dissolving the appropriate salts in deionized water (DIW) as outlined in Table 1.

**Table 1:** Composition of Permian Brine and Salts Used in Its Preparation

salt	concentration (M)	supplier	purity (%)
NaCl	2.007	Fisher Scientific	≥99.5
KCl	0.017		99.5
CaCl_2_·2H_2_O	0.027		99.0
MgCl_2_·6H_2_O	0.006	Sigma-Aldrich	≥99.0
SrCl_2_	0.005		≥99.0
NaBr	0.007		≥99.9
total ionic strength	2.070		

**Figure 1:**
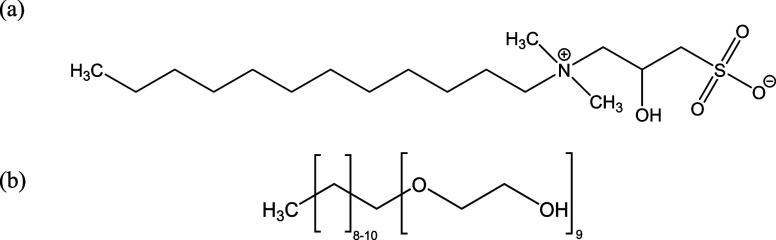
Structure of (a) alkyl hydroxysultaine: 3–dodecyldimethylammonio–2–hydroxypropane–1–sulfonate, (b) C_10–12_ nonaethylene glycol ether: C_10–12_E_9_.

**Figure 2:**
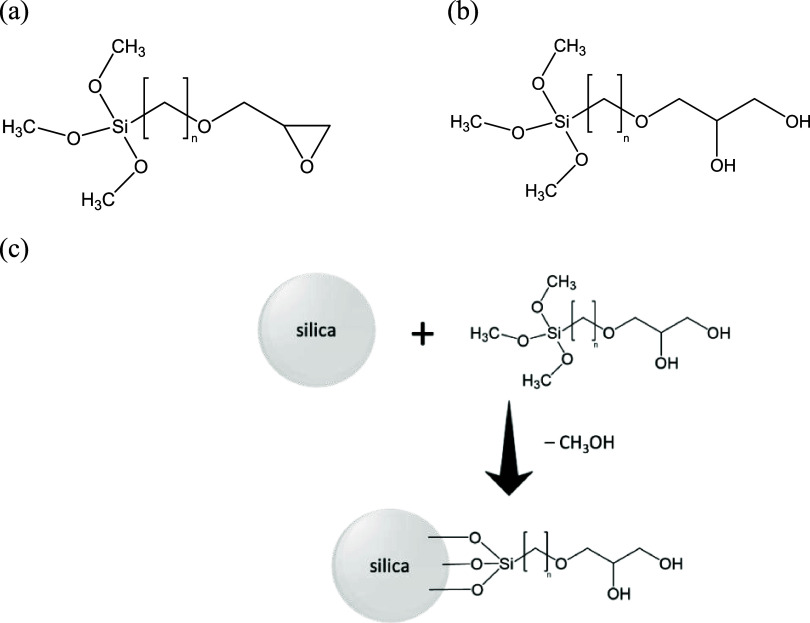
(a) Initial epoxysilane (ES), (b) hydrolyzed ES by HCl and (c) covalent grafting of hydrolyzed ES on a silica particle surface.

Table 2 shows the properties of two commercial surfactant solutions (Figure 1) and bare silica dispersion used in this study. An epoxysilane (ES) was used for surface modification of bare silica (Table 2 and Figure 2a). The properties of crude oil used in this study are also detailed in Table 3.

**Table 2:** Specifications of Chemicals Used in This Study

type	concentration (%)	supplier	comment
AHS surfactant solution	50	championX	aqueous zwitterionic alkyl hydroxysultaine
ZN surfactant solution	32		binary mixture of AHS and a minor amount (5 wt %) of nonionic C_10–12_ nonaethylene glycol ether (C_10–12_E_9_) blended with some proprietary additive
bare silica dispersion	30		pH ∼10, surface area of 331 m^2^ g^–1^
epoxysilane (ES)	–	Sigma-Aldrich	purity of ≥98%

**Table 3:** Properties of Crude Oil Sample Used in This Study

property	value	units
polar (resin)	1.4	%
aromatic	9.4	
paraffin	32	
asphaltene	<1	
total acid number^a^	0.25	mg KOH g^–1^
total base number^a^	0.45	
density at 25 °C^b^	0.8133	g cm^–3^

a Determined using potentiometric titration of Fan and Buckley^22^ by a T50 automatic titrator (Mettler Toledo, U.K.).

b Determined using a DMA 4500 density meter (Anton Paar, Austria).

Outcrop rock substrates and cores were used in this study. The rock was mainly calcite (97.2% calcite +2.8% quartz, Figure S1) and had a high Brunauer–Emmett–Teller (BET) surface area implying a high potential for adsorbing chemicals in EOR. The zero-point charge (ZPC) of rock in 0.01 M NaCl and Permian brine was measured to be ∼9.3 and ∼8.0, respectively (Figure S1). Table 4 presents the specifications of the cores and the solutions used in spontaneous imbibition and dynamic surfactant adsorption experiments.

**Table 4:** Specifications of the Cores Used for Dynamic Chemical Adsorption and Spontaneous Imbibition Tests^a^

dynamic chemical adsorption onto rock								
core no.	length (cm)	diameter (cm)	*V*_p_ (cm^3^)	*V*_b_ (cm^3^)	porosity (%)	permeability (md)	*S*_w_ (%)	comment
1	7.046	3.800	39.2	79.9	49.0	4.2 ± 0.4	–	0.01 wt % particles in Permian brine
2	6.740	3.810	38.2	76.8	49.8	4.3 ± 0.1		0.03 wt % AHS in Permian brine
3	7.056	3.802	39.5	80.1	49.4	3.6 ± 0.5		0.03 wt % AHS and 0.01 wt % particles in Permian brine
4	7.024	3.820	40.4	80.5	50.2	3.5 ± 0.3		0.05 wt % ZN in Permian brine
5	6.700	3.812	37.9	76.5	49.5	3.1 ± 0.2		0.05 wt % ZN and 0.01 wt % particles in Permian brine

spontaneous imbibition tests								
6	7.028	3.822	36.6	80.6	45.4	2.6 ± 0.8	10	secondary: Permian brine
								tertiary: 0.01 wt % particles +0.05 wt % ZN in Permian brine
7	7.080	3.826	38.2	81.4	47.0	3.7 ± 0.2		secondary: 0.05 wt % ZN in Permian brine
8	6.972	3.810	37.0	79.5	46.5	3.5 ± 0.4		secondary: 0.01 wt % particles +0.05 wt % ZN in Permian brine
9	7.000	3.812	36.3	79.9	45.5	3.0 ± 0.2		secondary: 0.03 wt % AHS in Permian brine
10	6.970	3.814	39.7	79.6	49.9	4.0 ± 0.7		secondary: 0.01 wt % particles +0.03 wt % AHS in Permian brine

a *V*p is the pore volume, *V*_b_ is the bulk volume, and *S*_w_ is brine saturation. 1 millidarcy (md) = 9.9 × 10^–16^ m^2^.

Sodium hydroxide (99%, Fisher Scientific) and hydrochloric acid (37%, Fisher Scientific) were used for pH adjustment where required. Sterile 33 mm diameter Startlab syringe filters with poly(ether sulfone) or hydrophilic polyvinylidene fluoride membranes were used for filtration (0.45 μm pore size).

### 2.2 Methods

#### 2.2.1 Particle Synthesis and Characterization

Chemical grafting was used to covalently adsorb silane onto the particle surface through a series of hydrolysis–condensation reactions (Figure 2). The extent of grafting was controlled by adjusting feed concentrations allowing us to optimize the synthesis for both aqueous colloidal stability and hydrophobicity. Different ES/silica particle ratios of 0.25, 0.50, 0.75, and 1.00 (g g^–1^) and a silane/HCl (0.01 M) ratio of 0.16 g g^–1^ were used in the synthesis. ES was first hydrolyzed using HCl for 10 min at 20 ± 2 °C (Figure 2a,b) and then added dropwise to the bare silica dispersion at room temperature for covalent grafting on particle surfaces (Figure 2c). The introduction of silane to the dispersion was performed at once or in incremental steps. In the stepwise approach, the dispersion was subjected to stirring after each silane addition followed by heating to 60 °C for a reaction period of 24 h. During the entirety of the process, the pH of the mixture was regulated to ∼10 using NaOH. Upon completion of the reaction, the resultant blueish dispersion was cooled to ambient temperature.

**Figure 3:**
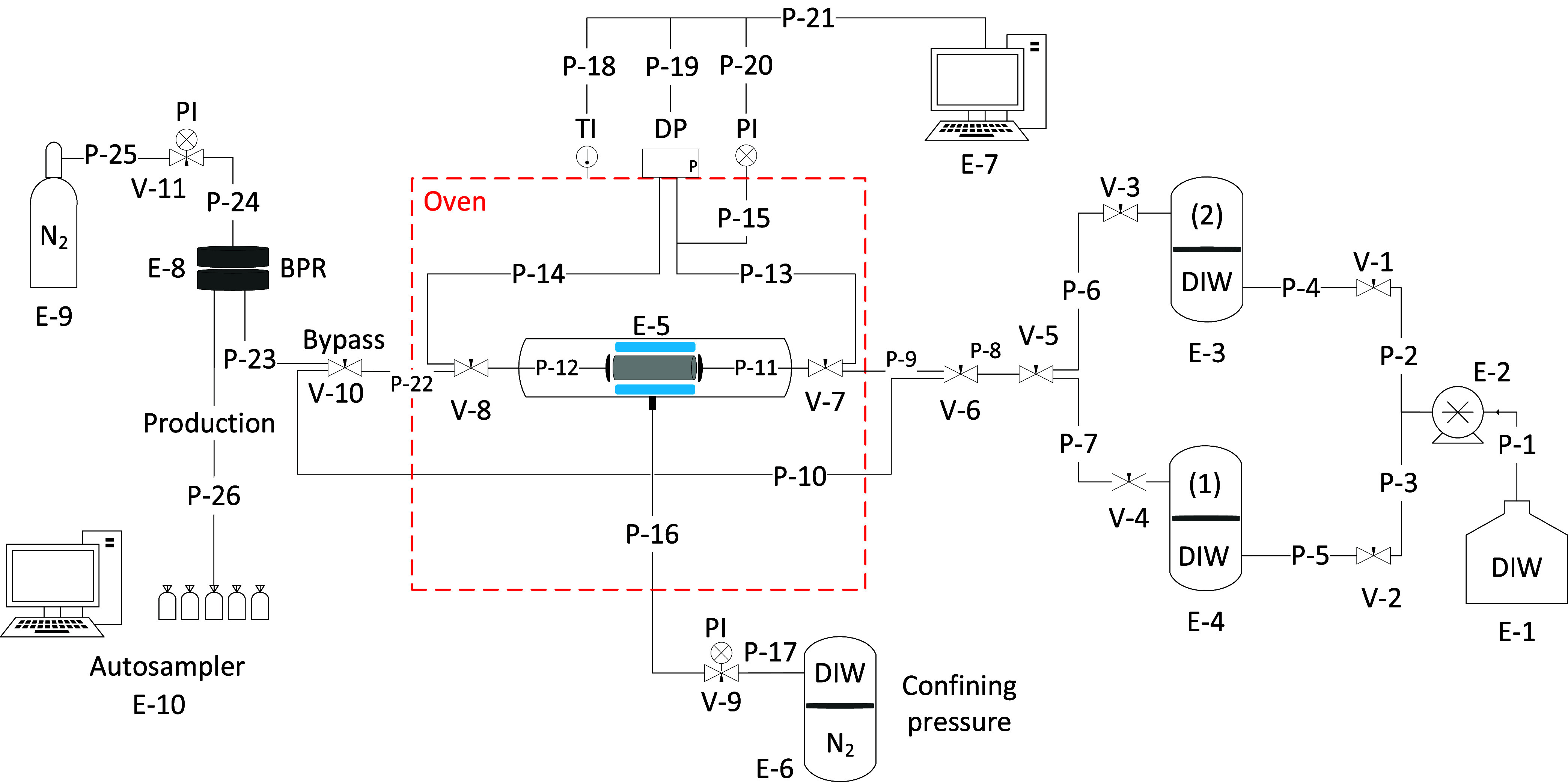
Scheme of core flooding setup. The letters P, V and E refer to pipelines, valves and equipment used in the setup, respectively. TI and PI are temperature and pressure indicators, respectively. DP is a differential pressure transmitter and BPR is a back pressure regulator. The red dashed box is the oven at 75 °C.

The diameter and ζ-potential of particles were measured at 25 °C using a Zetasizer Nano-ZS instrument (Malvern, U.K.). In addition to static stability assessment, particle stability was examined in cores to investigate potential particle aggregation under flow within porous media. The flooding setup included a Gilson 307 high-performance liquid chromatography (HPLC) pump (USA) with a DIW feeding bottle (E1 and E2), two transfer cylinders containing injection solutions or dispersions (E3 and E4), a Hasler core holder (E5), confining pressure by N_2_-pressurized DIW (E6), a Rosemount digital differential pressure transmitter (Emerson, U.K.) and thermometers (E7), a back pressure regulator (BPR) connected to a N_2_ source (E8 and E9), an autosampler (E10), an oven and appropriate tubing and valves (P1–P26 and V1–V11) (Figure 3). An empty clean core was mounted on the setup and flooded first by Permian brine (7 pore volume (PV)) and then ES-coated silica dispersion (3 PV) at 0.1 cm^3^ min^–1^ and 75 °C.

X-ray diffraction (XRD, using an Empyrean X-ray diffractometer) and thermogravimetric analysis (TGA, using a PerkinElmer TGA 4000) were performed on particles. Particle surface activity was assessed using air–water surface tension measurements at 25 °C using a K11 tensiometer (Krüss, Germany) and the du Nouy ring method. The measurements were repeated three times and the average was reported.

#### 2.2.2 Contact Angle Measurement

The rock substrates were polished using a polishing machine and silicon carbide suspensions (grit sizes #400 and #600). They were washed with DIW in a bath sonicator and dried at 90 °C for 1 day. They were then aged vertically in crude oil in separate glass vials for uniform hydrophobization at 20 ± 2 °C for a month. For the treatment process, the oil-wet substrates were placed vertically in solutions for up to 48 h at room temperature. The inverted sessile drop method using a DSA 10 instrument (Krüss, Germany) was used for oil–water contact angle measurements. At different treatment times (0.5, 1.5, 3, 24, and 48 h), multiple oil droplets (5 μL) were injected underneath the oil-wet substrate immersed in DIW or Permian brine using a U-shaped needle mounted on a Gastight 50 μL syringe (Hamilton). At least eight oil droplets were placed on both sides to account for the rock surface heterogeneity. The oil droplet profiles on the rock were photographed over time and equilibrium contact angles through water were determined using ImageJ software.

#### 2.2.3 Surfactant Adsorption Onto Rock

The extent of AHS or ZN adsorption onto rock powder and cores was analyzed with or without ES-coated silica in DIW and Permian brine. For static adsorption, 1 g of rock powder (140 μm) and 19 cm^3^ of solutions or dispersions were added to a glass vial and stirred magnetically in a water bath at 25 ± 0.5 °C for 24 h. The mixture was centrifuged for 10 min at 7500 rpm and the supernatant was collected using a syringe and passed through a 0.45 μm membrane filter to separate rock particles. Surface tension was used to determine the surfactant concentration in the supernatant. The process was repeated three times and the average with standard deviation was reported. The amount of surfactant adsorbed on rock (*Q*_e_) is given by^23^
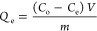
1where *C*_o_ and *C*_e_ are the initial and equilibrium surfactant concentrations, respectively (mg cm^–3^), *V* is the solution volume and *m* is the rock powder weight. The treated rock powder was dried in an oven at 90 °C for 24 h. A Zeiss EVO-60 scanning electron microscopy (SEM) instrument equipped with a LaB6 emitter at its second emission peak was used for SEM imaging from rock powder. An Oxford X-max 80 detector connected to Inca 1.2 software was used to gather spectra with a count rate of 1000–2000 counts/sec for 30 s in energy dispersive X-ray spectroscopy (EDS) for elemental analysis.

For dynamic adsorption, clean cores (see Section 2.2.6 for core preparation) were placed in a core holder (Figure 3) and flooded by Permian brine (7 PV) and surfactant solutions with or without particles (5 PV) at 0.1 cm^3^ min^–1^ and 75 °C with a back pressure of 10 bar and a confining pressure of 18 ± 1 bar. Injection pressures, differential pressures and temperatures were recorded throughout the experiment. Sampling was performed from the produced liquid every 80 min for air–water surface tension measurements at 25 °C.

#### 2.2.4 Fluid-Fluid Interfacial Tensions

Air–water surface tension was measured at 25 °C using the du Nouy ring method. Oil–water interfacial tension measurements were performed using a Site 04 spinning drop tensiometer (Krüss, Germany) at 25 and 60 °C. In both experiments, measurements were repeated three times and the average was reported.

#### 2.2.5 Emulsions

Five g of oil and 5 g of solutions or dispersions were gently added to a glass vessel and homogenized using a T25 digital ultraTurrax homogenizer (IKA, China) for 2 min at 13,000 rpm at room temperature. An emulsion drop test was performed for the determination of emulsion type. Initial microscope images of the emulsion were taken using an Olympus BX51 microscope fitted with a GXCAM U3–18 digital camera. The emulsion droplet diameter D[4,3] was determined using a Mastersizer 2000 instrument (Malvern, U.K.). The stability of emulsions was monitored with time at room temperature from photos, and the fractions of oil (*f*_o_) and water (*f*_w_) resolved were calculated based on the following
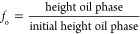
2
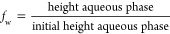
3

#### 2.2.6 Spontaneous Imbibition Tests

The efficiency of formulations in oil recovery was evaluated through spontaneous imbibition tests. For this, the cores were first cleaned by flooding with DIW for 5 PV at 0.1 cm^3^ min^–1^ at room temperature to remove sulfate (confining pressure of 20 ± 2 bar). The absolute permeability of the cores was then calculated using the Darcy equation at 0.05, 0.10, and 0.15 cm^3^ min^–1^. The dried cores were then vacuumed for 15 min to a pressure of 0.3 mbar in a desiccator and saturated with 10-time diluted Permian brine (ρ = 1.01 g cm^–3^ at 25 °C) for 30 min by which the pore volume (*V*_p_) and porosity of the cores were estimated (see Table 4). The brine-saturated cores were conditioned in a desiccator with silica gel to reach a brine saturation of 10%. They were then placed in sealed plastic containers at room temperature for 3 days to ensure a homogeneous distribution of brine in the rock. A total of 5 PV of fresh crude oil was then injected into the cores for oil saturation. For this, the cores were first mounted on a core holder (Figure 3) and vacuumed for 1 h until stabilizing at 0.3 mbar. They were first pressurized to 5 bar by 1 PV injection of fresh crude oil at 50 °C at 0.5 cm^3^ min^–1^ for uniform oil saturation. Two PV crude oil was then injected into each side of the cores at 0.2 cm^3^ min^–1^ at 50 °C. A small amount of crude oil was finally injected into the cores at room temperature to account for the rock expansion at high temperatures. The oil-saturated cores were then aged in high pressure high temperature (HPHT) cells at 75 °C for 2 weeks to reduce water-wetness. For spontaneous imbibition tests, the cores were placed in HPHT imbibition cells connected to a transfer cylinder containing 1 L of the imbibition fluid pressurized to 10 bar using N_2_. The oil production from the spontaneous imbibition process at 75 °C was recorded daily. The imbibition tests were performed in secondary and tertiary modes.

## 3 Results and Discussion

First, the properties of surfactants, synthesized particles and their mixtures are presented as the basis for further experiments. Next, the study examines particle-surfactant mixtures at the rock-water interface focusing on rock wettability alteration and surfactant adsorption supported by microscopy and elemental analysis. Then, the impact on oil–water interfacial tension and emulsification is evaluated. Finally, spontaneous imbibition tests assess the efficiency of candidate blends in porous media.

### 3.1 Surfactant Properties

Surfactant properties determine its bulk and interfacial behavior in EOR applications. In this study, surfactants showed no precipitation or degradation (up to 150 °C, less than typical reservoir temperatures) in Permian brine after standing at 100 °C for one month (Figure S2). The AHS surfactant showed a net zero charge at a pH of 5.5–8.0 at 25 °C while this region was narrower for ZN (pH = 7.5–8.4) as shown in Figure S3. The CMC of AHS in DIW and Permian brine at 25 °C was 0.01 and 0.005 wt %, respectively, while that of ZN was 0.02 and 0.003 wt %, respectively. Zwitterionic surfactants are weakly anionic even at zero net charge pH for two reasons. First, the negative charge of the headgroup is located on the end of the molecule and the positive group is in the middle part leading to a higher charge density in the negative part. Second, it is mostly the anionic group that interacts with inorganic cations in micellar form. Thus, even at neutral charge pH, electrostatic contribution contributes to the reduction of the CMC of zwitterionic and nonionic surfactants in addition to salting out effect.^24^

### 3.2 Nanoparticle Properties

Nanoparticles were synthesized using different feed concentrations and assessed through various characterization tests including particle size, ζ-potential, XRD, TGA-DTG, air–water surface tension and colloidal stability in high salinity brine. Although further techniques like ultraviolet–visible (UV–vis) and Fourier transform infrared (FTIR) spectroscopy could have been applied,^25^ they were unsuccessful due to chemical interference. However, the techniques we employed were effective in characterizing the nanoparticles.

#### 3.2.1 Effect of Silane Mass

Nanoparticle stabilization is crucial for their effective utilization in EOR. The synthesis of silane-coated silica here was performed using different silane amounts to investigate its effect on final surface coverage and thus particle stability. Regarding the synthesis type, one-step synthesis was the least effective approach since it may provide silane–silane condensation reducing effective covalent grafting of silane on particles. The most effective approach was determined to be a two-step addition of silane since four-step synthesis did not improve stability noticeably. A full silane monolayer on silica was found to be at 0.6 g g^–1^ assuming a monodentate particle-silane interaction. A rise in initial particle diameter in DIW and Permian brine at 25 °C was observed for silane/silica ratios above 0.75 g g^–1^ due to particle aggregation because of interactions between adsorbed silane on silica and excess silane (Table 5). The stability evaluations revealed that a silane/silica ratio of 0.35 g g^–1^ is the optimal silane coverage for ensuring long-term colloidal stability under high salinity and high temperature conditions. This optimal silane coverage was used consistently throughout the entirety of this study.

**Table 5:** Effect of ES Mass Used during the Two-Step Synthesis of ES-Coated Silica on the Initial Particle Diameter in DIW and Permian Brine at 25 °C^a^

	d/nm	
mass ES per g silica/g	DIW	Permian brine
0.25	20 ± 3	23 ± 1
0.50	20 ± 2	23 ± 1
0.75	23 ± 1	28 ± 3
1.00	120 ± 3	450 ± 50

a [Particle] = 0.1 wt %.

#### 3.2.2 Surface Chemistry

The particle surface chemistry determines its bulk and interfacial interactions in EOR applications. The surface chemistry of optimized ES-coated silica was assessed here by XRD and TGA. The XRD indicated additional peaks confirming the grafting of silica with silane by covalent bonds (Figure S4). TGA on particles showed no significant weight loss when heating the particles from 110 to 200 °C indicating that the silane can tolerate the typical reservoir temperatures (70–130 °C)^26^ (Figure S4). The silane coverage on silica for these particles was calculated at 55% of a full monolayer (see eq S1). The particles caused a minor reduction in air–water surface tension implying low surface activity (Table S2).

#### 3.2.3 Colloidal Stability

Bare silica (see Table S3: *d* = 15 ± 0.5 nm, ζ = −43.8 ± 1.2 mV for 0.1 wt % in DIW) was found to lose its stability in Permian brine at 75 °C within just a few hours. Additional colloidal stability up to 1 week was achieved in Permian brine at 75 °C by 55% silane coverage (Figure S5) resulting in a relative rise in particle diameter and ζ-potential (see Table S3: *d* = 20 ± 0.5 nm, ζ = −26.9 ± 0.6 mV for 0.1 wt % in DIW). However, this stability improvement was not enough for practical EOR applications. Significant stability enhancement for more than 6 months was observed through further electrostatic stabilization by pH adjustment (Figure S5) with no effect on initial particle diameter.

**Figure 4:**
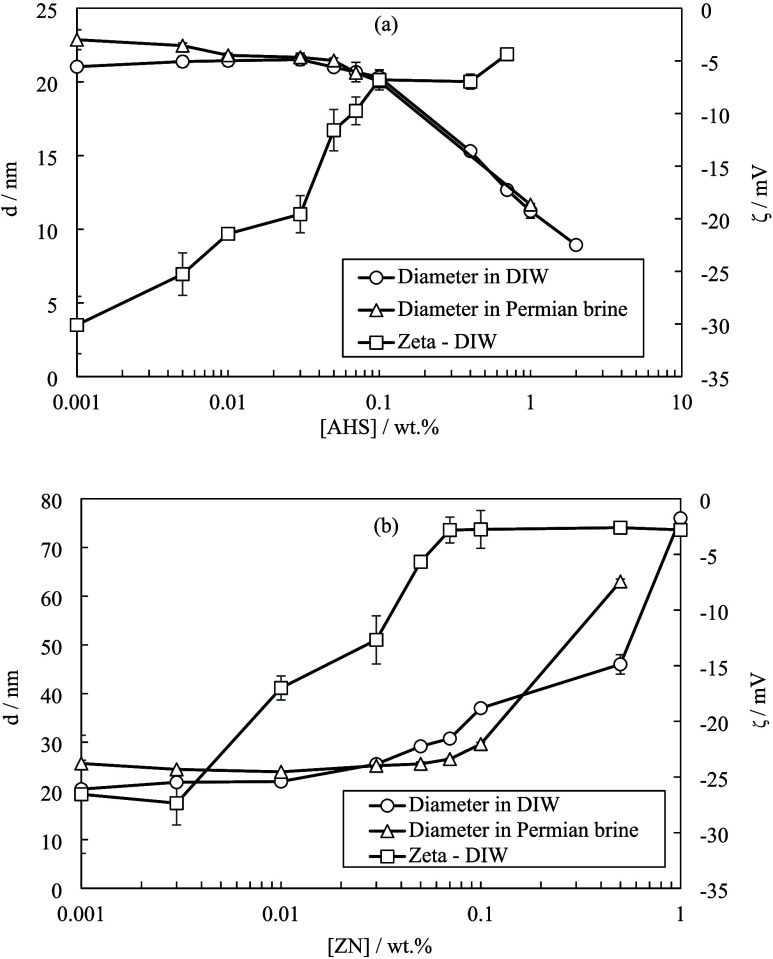
Initial particle diameter and ζ-potential of 0.1 wt % ES-coated silica in a blend with different concentrations of (a) AHS and (b) ZN in DIW and Permian brine at 25 °C.

Particle stability was also inspected for blends of ES-coated silica and surfactants (Figure S6). The quaternary ammonium headgroup of the zwitterionic surfactant can electrostatically adsorb onto the anionic nanoparticle surface provided that the sulfonate group bends for V adsorption, leaving the hydrocarbon tails toward the water phase. This physical grafting was observed to increase particle colloidal stability for up to 4 weeks at 75 °C by providing additional steric stabilization. It is noted that the same physical grafting can happen between bare silica and zwitterionic surfactant but it is most likely that their electrostatic attraction is screened by ions causing particle aggregation. This highlights the importance of chemical grafting of particles in EOR applications. Figure 4 shows the initial particle diameters in the blends. It indicates that particle diameter decreases with an increase in AHS concentration probably due to the increased electrostatic adsorption of surfactant molecules on particle surfaces which agrees with the increased ζ-potential. For blends with ZN surfactant, high surfactant concentrations (0.5 and 1 wt %) caused sedimentation at 75 °C after a day (Figure S6) probably due to the van der Waals forces between nonionic C_10–12_E_9_ tails adsorbed on particles.

**Figure 5:**
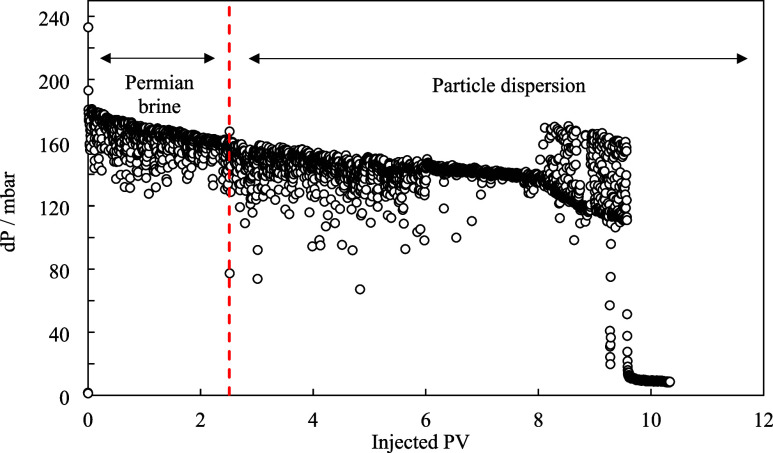
Differential pressure versus injected pore volume for a dried core injected with Permian brine followed by 0.01 wt % ES-coated silica particles in Permian brine at 0.1 cm^3^ min^–1^ and 75 °C. The error bar is ±3 mbar.

Different colloidal stability inspections can be conducted on synthesized particles.^27^ In addition to static particle stability, the synthesized nanoparticles were found to maintain stability while moving through porous media. There was no indication of significant particle aggregation (no continuous pressure buildup) during dispersion injection, as shown in Figure 5 (see also Figure S7). However, particle addition to the injected phase produced larger pressure fluctuations within porous media. The particles may temporarily reduce permeability due to the localized log-jamming and pore throat obstruction which can reroute the injected phase into adjoining oil-filled channels for more EOR.

### 3.3 Surfactant and Particles at Rock-Water Interface

#### 3.3.1 Oil–Water Contact Angles

##### 3.3.1.1 Particles

Certain nanoparticles have the potential to modify rock wettability from oil-wet to water-wet. To inspect this, oil–water contact angle measurements were made for oil-wet substrates treated with particle dispersions. As shown in Table S4, the largest contact angle reduction by ES-coated silica alone was 30° which was less than that of bare silica. Consistent with their low surface activity at the air–water surface (see Table S2), ES-coated silica was found surface inactive on rock for wettability alteration. Their limited ability to reduce the oil–water contact angle is consistent with earlier research findings which indicated that particles grafted with nonionic ligands are less effective than ionic ones in changing calcite wettability.^28^ The high silane coverage on particles here increases their hydrophilicity through hydroxyl groups on ES but results in significantly prolonged and improved dispersion stability in high salinity Permian brine, a characteristic not observed in previous studies.^29^ The observed oil–water contact angles with the particles here highlight the imperative need to introduce surfactants for a discernible rock wettability alteration for EOR purposes.

##### 3.3.1.2 Surfactants

**Figure 6:**
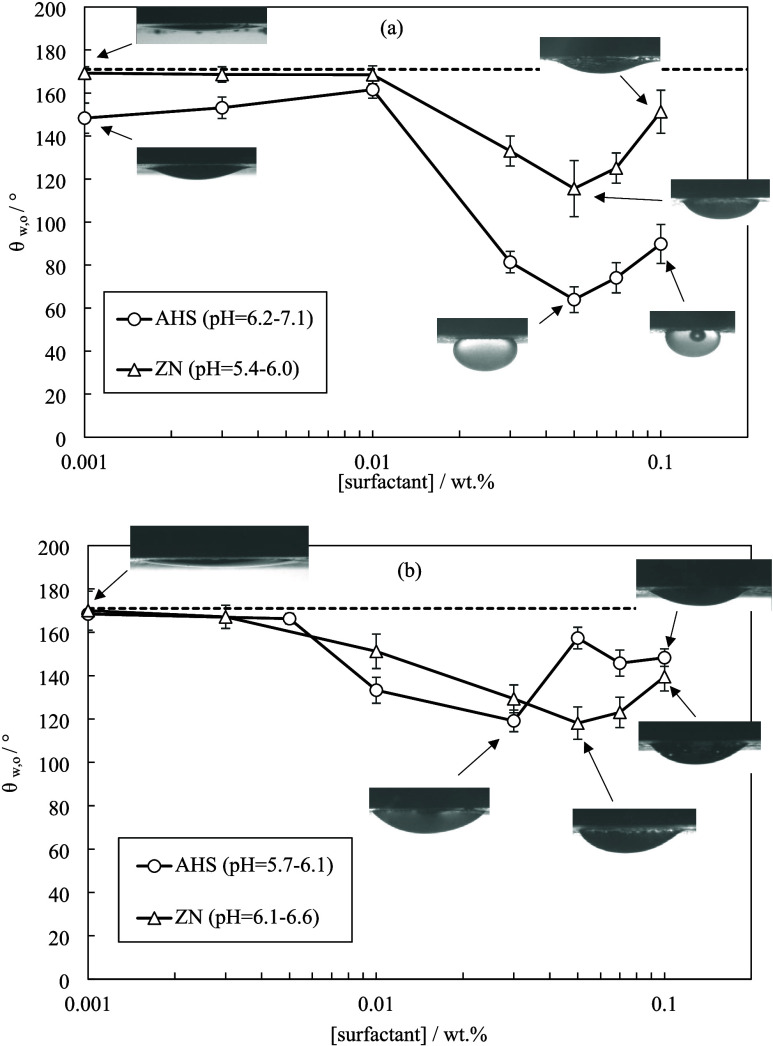
Equilibrium contact angles of crude oil droplets in water on oil-conditioned rock treated for 24 h with different concentrations of ZN or AHS in (a) DIW and (b) Permian brine. The dashed line signifies contact angle of oil droplets in Permian brine.

**Figure 7:**
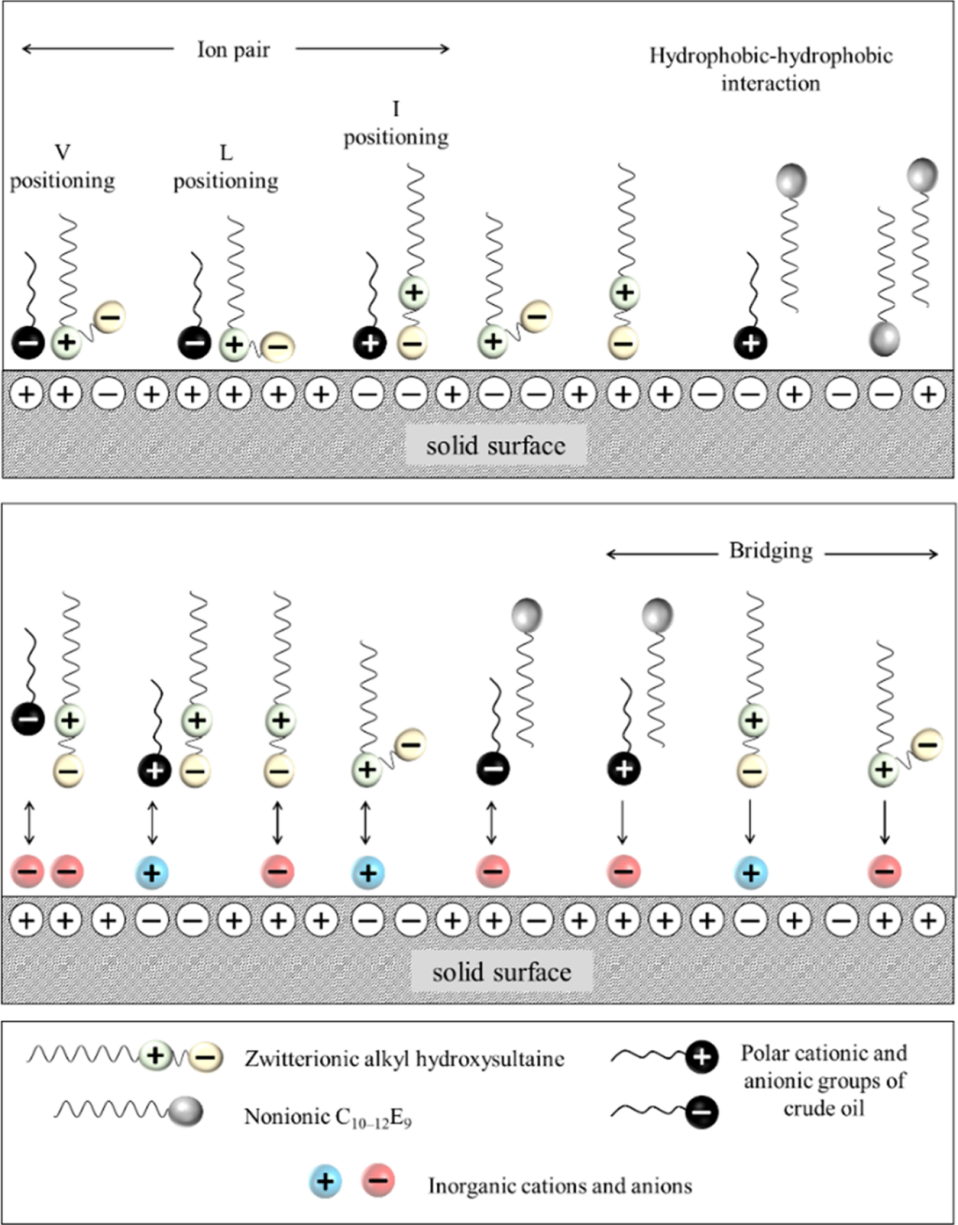
Schematic representation of the wettability alteration of calcite-rich rocks by zwitterionic and nonionic surfactant molecules in the absence and presence of inorganic salts.

Surfactants are well-known for their ability to alter rock wettability in EOR processes. When surfactants were in DIW, rock wettability alteration was more noticeable for AHS compared to ZN (Figure 6). The ammonium headgroup of zwitterionic surfactant can adsorb on anionic sites on the rock surface by V or L adsorption depending on the nearby rock charge interacting with the sulfonate group (Figure 7). The L position occupies a larger surface area and leads to a lower adsorption density. On the other hand, the rock cationic sites attract the anionic sulfonate headgroup of the surfactant and tend to be away from the cationic ammonium group resulting in normal I adsorption with a lower adsorption area per molecule and thus a higher adsorption density on the rock (Figure 7). In this study, since the rock was mostly calcite and net cationic, I-shaped adsorption is expected to be dominant.

With both positive and negative charges in their headgroup, zwitterionic surfactants can also electrostatically attract various natural ionic surfactants from crude oil that are adsorbed on the rock surface. This attraction leads to the formation of ion pairs facilitating the desorption of oil components from the rock surface leading to rock wettability alteration from oil-wet to water-wet (Figure 7). These desorbed components can then form mixed micelles in bulk. Strong H-bonds between surfactant headgroups and the rock surface are also possible for the nonionic C_10–12_E_9_ present in ZN given that it has a high ethylene oxide number (high hydrophilicity), allowing the hydrophobic tail of the surfactant to be oriented outward for additional hydrophobic–hydrophobic interaction with free surfactant molecules to create a hydrophilic bilayer on the rock surface (Figure 7) rendering the rock surface water-wet.

Ions can change the adsorption behavior of surfactants. Figure 6 shows the contact angle measurements for surfactants in Permian brine. The addition of Permian salts increased the minimum contact angle that could be achieved by AHS (from 64 ± 6 to 119 ± 5°) but it had a negligible effect on that of ZN. The AHS concentration for the minimum contact angle was lower than that of ZN (0.03 versus 0.05 wt %). The ions could lower the electrostatic attraction between surfactant headgroups and the solid surface (or ionic oil components) and thus reduce the surfactant efficiency in rock wettability alteration.^30^ This may account for the higher oil–water contact angles by AHS in the presence of Permian brine here, consistent with the reduced AHS adsorption on the rock on adding brine (Figures S9 and S10). High electrolyte concentrations can increase the chemisorption of counterions on reactive sites of solid surfaces which makes nonionic surfactants ineffective for solid wettability alteration here.^31^

##### 3.3.1.3 Surfactant-Particle Mixture

Surfactants and nanoparticles may adsorb onto rock through different mechanisms. They can form weak van der Waals interactions facilitating initial binding. Given that the calcite surface is charged, surfactants with oppositely charged headgroups can strongly adsorb onto rock *via* electrostatic attraction. Additionally, surfactant headgroups may form hydrogen bonds with hydroxyl groups on the calcite surface enhancing interaction stability. Surfactants can also modify the surface energy and wettability of nanoparticles enhancing their hydrophobicity for particle adsorption onto rock.^8,21^

The contact angle measurements with blends of surfactant and ES-coated silica in DIW are presented in Figure S8. A further reduction in contact angle was observed when combining particles and surfactants compared to the effects of particles or surfactant alone. This suggests a synergistic effect likely arising from enhanced surfactant adsorption onto the rock surface due to electrostatic repulsion by particles.

**Figure 8:**
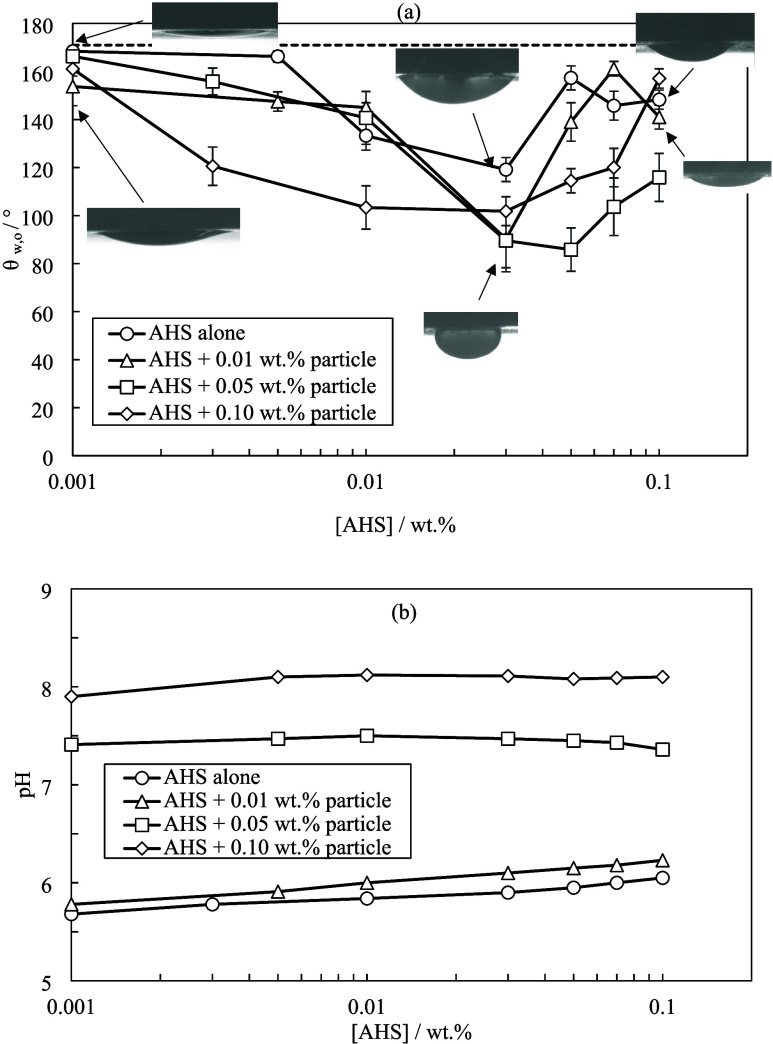
(a) Equilibrium contact angles of crude oil droplets in water on oil-conditioned rock treated for 24 h with different [AHS] in Permian brine with or without ES-coated silica at different concentrations. The dashed line signifies the contact angle of oil droplets in Permian brine. (b) pH values of blends at 20 ± 1 °C.

Figure 8 shows the oil–water contact angle and pH measurements for blends of AHS and ES-coated silica in Permian brine. In comparison with surfactant alone, a reduction of 30 and 72° is observed at 0.03 and 0.05 wt % AHS on the addition of 0.01 and 0.05 wt % particles, respectively. These further contact angle reductions agree with the increased AHS adsorption onto rock due to the addition of particles to Permian brine (Figures S9 and S10). Permian brine provides high surfactant adsorption onto rock and particles with tails pointing outward which promotes hydrophobicity of both surfaces. A rise in pH causes the surfactant to become weakly anionic which develops electrostatic repulsion between anionic particles and surfactant that promotes the adsorption of surfactant on the rock and induces further wettability alteration compared to AHS alone.

**Figure 9:**
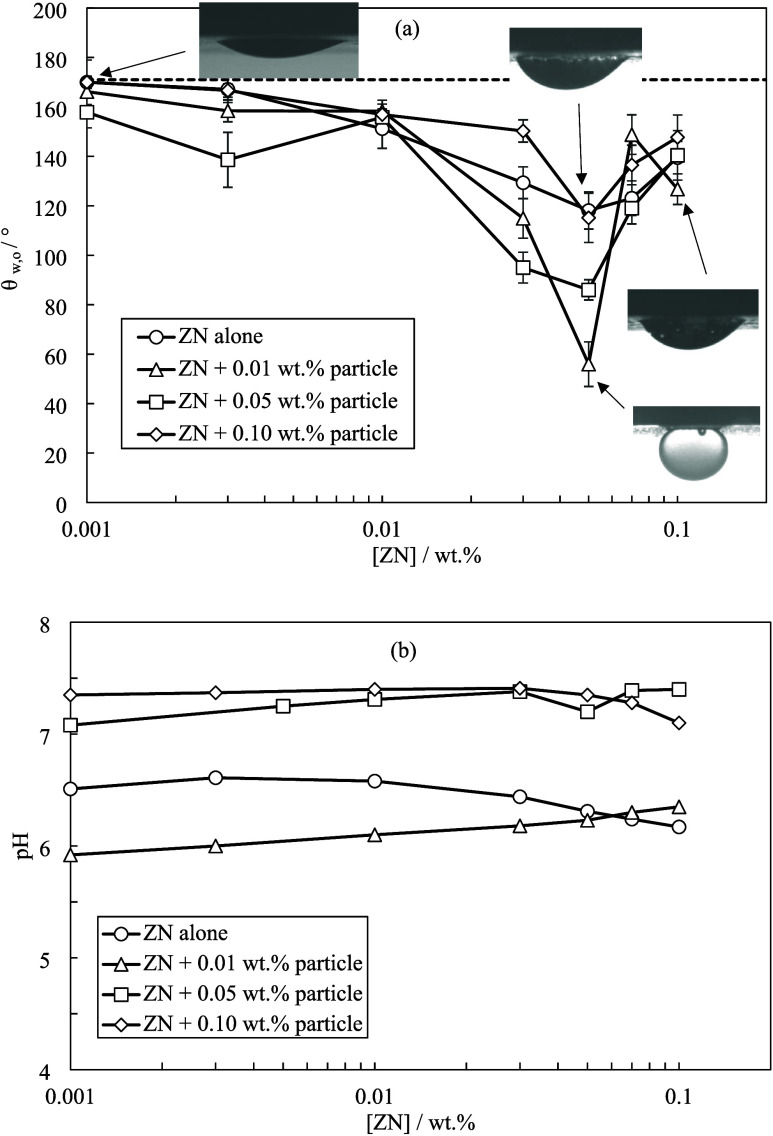
(a) Equilibrium contact angles of crude oil droplets in water on oil-conditioned rock treated for 24 h with different ZN concentrations in Permian brine with or without ES-coated silica at different concentrations. The dashed line signifies the contact angle of oil droplets in Permian brine. (b) pH values of blends at 20 ± 1 °C.

Figure 9 shows the oil–water contact angle and pH measurements for blends of ZN surfactant and ES-coated silica in Permian brine. There is a minimum contact angle at 0.05 wt % ZN at different particle concentrations. The lowest contact angle (56 ± 5°) is observed with 0.01 wt % particles and 0.05 wt % ZN in Permian brine which resulted in an additional reduction of 62° compared to that of surfactant alone at the same concentration. Furthermore, the addition of Permian brine to blends of ZN and particles reduced the optimum surfactant concentration from 0.07 to 0.05 wt %. It is thought that the adsorption of surfactant molecules on the surface of particles makes them partially hydrophobic and surface-active. This agrees with the reduced ZN adsorption onto the rock with the addition of particles in Permian brine (Figures S11 and S12). However, high surfactant concentrations can increase the hydrophobicity of particles which is consistent with the increase in ζ-potential upon increasing surfactant concentration. The contact angle results of particle-surfactant blends in Permian brine identify the following blends as optimum formulations for EOR:

- 0.01 wt % particles and 0.03 wt % AHS surfactant in Permian brine
- 0.01 wt % particles and 0.05 wt % ZN surfactant in Permian brine

##### 3.3.1.4 Effect of pH

Table 6 shows the effect of pH on the performance of blends of ZN and ES-coated silica in Permian brine on rock wettability alteration. The charge of surfactant, particles and rock is affected by pH as is their interactions. Below and above its net zero charge region, ZN transitions into cationic and anionic, respectively (Figure S3). In addition, bare silica surface charge decreases with a decrease in pH toward its isoelectric point (pH 2–3). A lower surface charge is expected for ES-coated silica due to the silane coverage. Accordingly, three possible interactions can occur:

- Low pH (4): Weak electrostatic attraction between cationic AHS and weakly anionic particles or crude oil carboxylates (p*K*_a_ = 4) exists. The adsorption of cationic AHS on cationic calcite is also negligible. Although expected to be helpful, calcite dissolution at low pH has not improved the rock wettability alteration probably due to the newly created surface serving as a new adsorbent for crude oil components or excess aqueous surfactant.
- High pH (>8.5): Weak electrostatic attraction between the ammonium headgroup of surfactant and anionic particles or crude oil carboxylates is expected. In this case, the particles may electrostatically repel the anionic surfactant causing it to adsorb excessively on the rock resulting in hydrophobicity again as observed before (Figure 9).
- Zwitterionic pH (7 ± 1): Intermediate pH is the optimal pH where the surfactant is zwitterionic (both charges exist) and particles are moderately charged resulting in the partial hydrophobization of particles by the surfactant and electrostatic attraction between crude oil components and particles or surfactant which have a significant effect on rock wettability alteration (Figure 9).

**Table 6:** Effect of pH on Contact Angles of Crude Oil Droplets in Water on Oil-Conditioned Rock Treated for 24 h with Dispersions of 0.1 wt % ES-Coated Silica and ZN Surfactant in Permian Brine at Original and Reduced pH (by HCl)

		θ_w,o_/°	
[particle] (wt %)	[ZN] (wt %)	original pH (7.1–7.4)	reduced pH (4.0)
0.1	0.001	170 ± 3	167 ± 3
	0.003	167 ± 3	169 ± 3
	0.01	157 ± 4	165 ± 6
	0.03	150 ± 5	168 ± 7
	0.05	115 ± 10	168 ± 7
	0.07	136 ± 8	159 ± 11
	0.1	148 ± 9	119 ± 10

#### 3.3.2 Surfactant Adsorption Onto Rock

**Figure 10:**
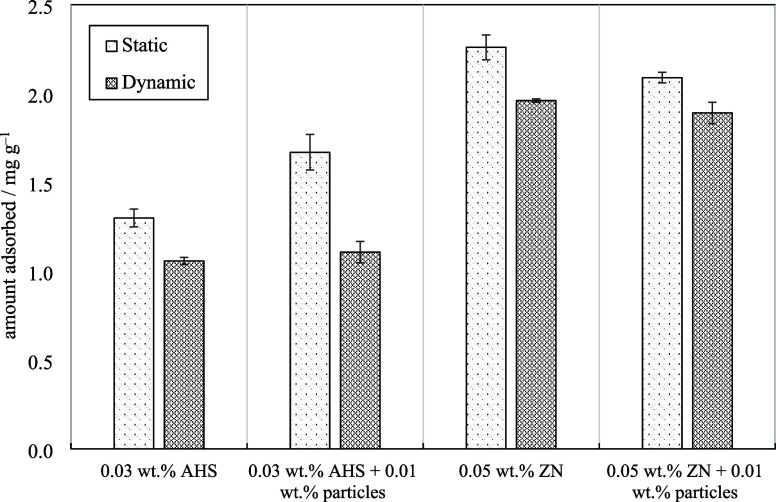
Adsorbed surfactant values onto rock powder (static adsorption at 25 °C) and porous media (dynamic adsorption at 75 °C) for AHS and ZN solutions with and without 0.01 wt % ES-coated silica particles.

Minimum surfactant adsorption on rock with the highest wettability alteration is always a target in EOR processes. The static surfactant adsorption analysis is presented in Figures S9–S12. The experimental data best fit with the Redlich-Peterson model (see model parameters in Table S5). Figure 10 compares static and dynamic surfactant adsorption onto rock for two surfactant solutions. The order of surfactant adsorption on rock surfaces with or without particles is ZN > AHS. The findings indicate that the addition of particles enhances the adsorption of AHS onto the rock while decreasing the adsorption of ZN. The changes are more pronounced in static mode. The composition of surfactants is the primary cause of their varying adsorption behaviors. Anionic particles and zwitterionic AHS have less electrostatic attraction when exposed to Permian brine ions. The same effect is expected to happen with ZN since it mainly consists of AHS but the interactions between the headgroup of the nonionic surfactant of ZN and the particle surface are independent of the salinity leading to more ZN adsorption onto particles and thus less adsorption onto rock. The ZN savings by particles are not very noticeable because of the low concentrations of both the nonionic surfactant and the particles. Surfactant adsorption at different surfaces decreases as follows: oil–water > particle–water > air–water. Therefore, the surfactant molecules tend to stay adsorbed on the particle surface in an air–water system but spontaneously leave the particle surface close to the oil–water interface.

#### 3.3.3 SEM-EDS

**Figure 11:**
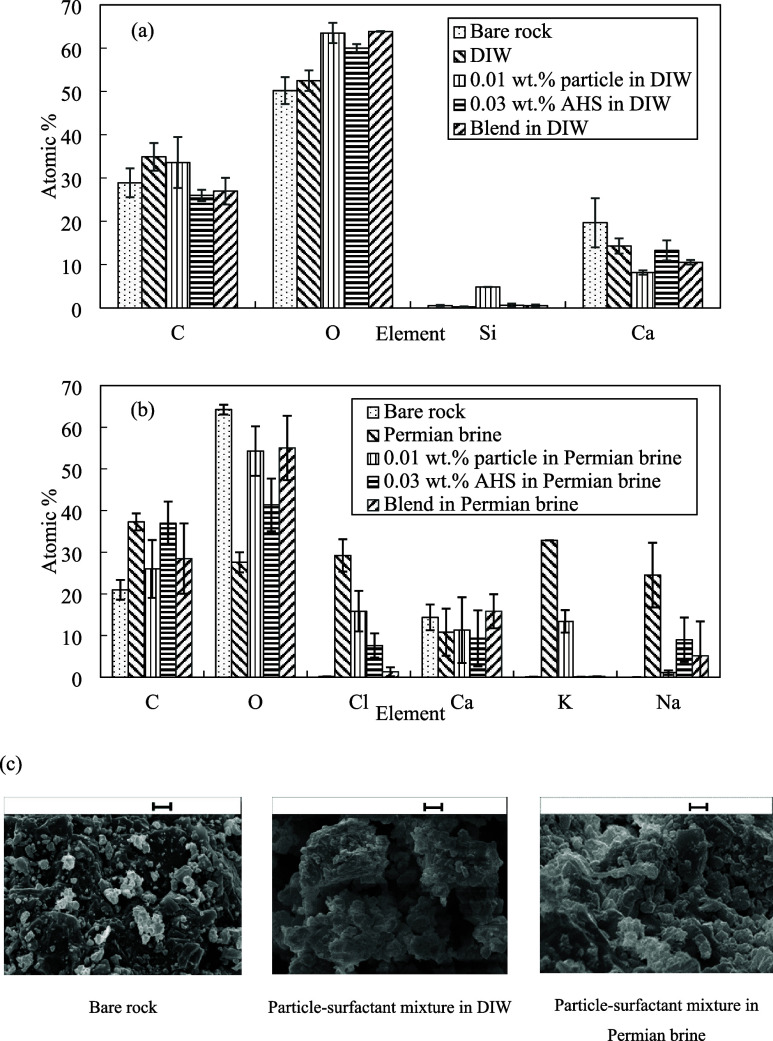
(a, b) EDS analysis, (c) SEM images taken from 10 μm rock powder after 24 h treatment with AHS solutions with and without ES-coated silica in DIW and Permian brine at 25 °C. Scale bar = 2 μm.

SEM-EDS analysis was performed on rock treated with various chemicals to provide visual insights and elemental analysis regarding rock wettability alteration. As shown in Figure 11, treating the rock with 0.01 wt % ES-coated silica in DIW results in a 10-fold increase in the percentage of surface silicon atoms compared to that of DIW alone (from 5%). This increase suggests the adsorption of particles on the rock surface accompanied by a higher number of oxygen atoms and a lower number of calcium atoms compared to bare rock. A relative rise in oxygen percentage compared to that of bare rock is observed after treatment with AHS solution, probably due to the adsorption of the sulfonate group on the rock, which is accompanied by a drop in calcium percentage due to the reduction of adsorption sites. By treating the rock with a blend of particles and surfactant, the percentage of surface oxygen is as high as the particle dispersion or surfactant solution and no rise in the number of silicon atoms as a result of particle adsorption on a solid surface is observed compared to that of bare rock probably due to the interference caused by the surfactant-particle interactions. The number of calcium atoms decreases when treating the rock with the blend compared to bare rock.

The addition of Permian salts reduced the oxygen percentage but left the calcium and carbon numbers almost unchanged. The percentage of chlorine, sodium and potassium ions decreased when the rock was treated with particles in brine probably due to the adsorption of ions onto the particle surface. When treating the rock with the surfactant solution, the percentage of carbon and oxygen increased and decreased, respectively compared to that of bare rock as a result of surfactant adsorption onto the rock while a further decrease in the percentage of the three brine ions (chlorine, sodium and potassium) was observed possibly due to the ions serving as counterions for surfactant headgroups. These elemental variations agree with surface morphological changes observed in SEM images upon the addition of ions, particles and surfactant (Figure 11).

### 3.4 Surfactant and Particles at Oil–Water Interface

**Figure 12:**
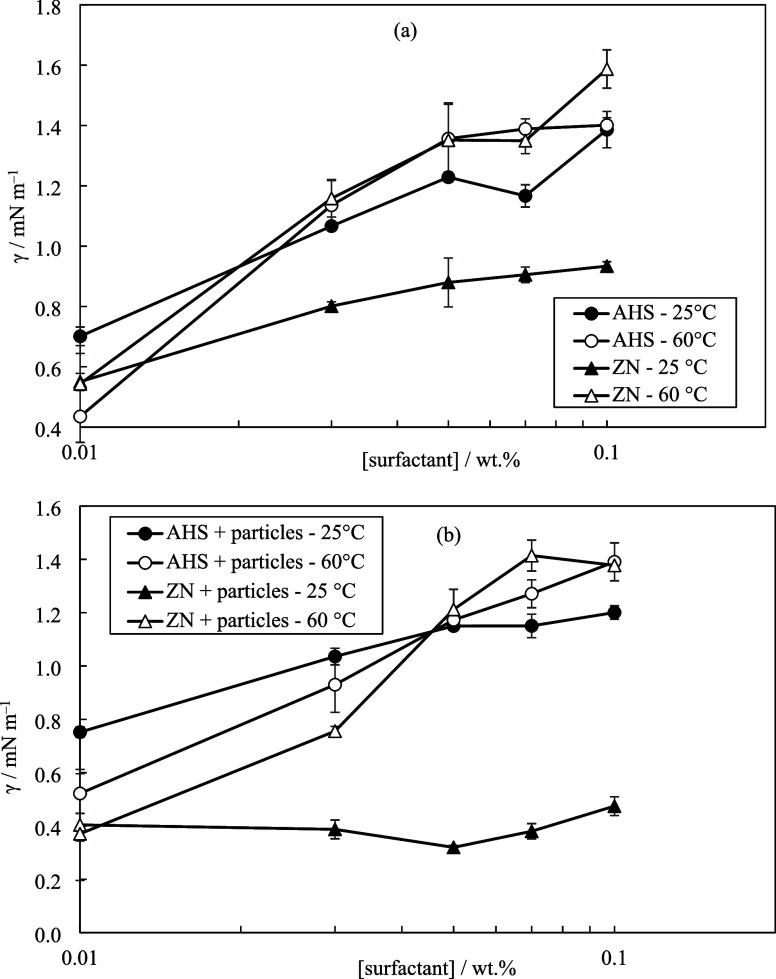
Effect of temperature on the interfacial tension between crude oil and water for different concentrations of (a) surfactant alone, (b) surfactant +0.01 wt % ES-coated silica in Permian brine.

Surfactants exhibit a unique capability to facilitate the movement of oil droplets by lowering the oil–water interfacial tension. The interfacial tension between crude oil and DIW at 25 °C was measured here to be 10.4 mN m^–1^ which was increased to 12.6 mN m^–1^ on adding Permian brine. A significant interfacial tension reduction was achieved at 25 °C upon adding surfactant (Figure 12) due to the mixed monolayer formed at the oil–water interface by added and indigenous surfactants. As surfactant concentration increases, both surfactants show a rise in interfacial tension likely due to bulk surfactant aggregation. Since post-CMC surfactant concentrations are utilized, increasing surfactant concentrations lead to bulk aggregation once the oil–water interface is saturated. This phenomenon results in increased solubilization of crude oil natural surfactants, depleting the oil–water interface and subsequently increasing interfacial tension. This was evidenced by the brownish color of the aqueous phase after contacting oil and water phases. The effect of temperature on AHS (with and without particles) in interfacial tension alteration was seen to depend on surfactant concentration. A significantly higher interfacial tension was achieved upon increasing the temperature at all ZN concentrations with and without particles in Permian brine.

Some researchers state that zwitterionic surfactants typically perform better as temperature rises because of their increased solubility and diffusion coefficient which can result in both lower interfacial tensions and shorter times to achieve a minimum interfacial tension.^32,33^ Some authors have reported increased oil–water interfacial tension on increasing temperature when using crude oil and brine.^34^ The hydration of the zwitterionic surfactant headgroups can be destroyed by high concentrations of counterions (Ca^2+^ or Na^+^). This causes the surfactant to transfer from the oil–water interface to the oil phase, progressively increasing the interfacial tension. This effect is more pronounced at high temperatures.^34^ On the other hand, temperature elevation weakens the solvation of surfactant molecules at the oil–water interface which consequently increases the interfacial tension.^35^ The larger slope in the plot of ZN may be related to the additional rise in interfacial tension caused by the nonionic surfactant present in ZN. This is attributed to the reduced solubility of ethylene oxide in water at high temperatures.

The type of particles and their interactions with surfactant or the oil–water interface are also affected by temperature. On one hand, temperature rise can reduce the effective surface area of particles and cause particle aggregation, both of which affect their adsorption behavior. The latter is absent here as the particles were found to be long-term stable in Permian brine at higher temperatures (75 °C). On the other hand, an increase in temperature has the potential to boost particle adsorption displacing adsorbed natural surfactants of crude oil from the oil–water interface toward the oil phase. This, in turn, results in an elevation of oil–water interfacial tension.^36^

### 3.5 Spontaneous Imbibition Tests

**Figure 13:**
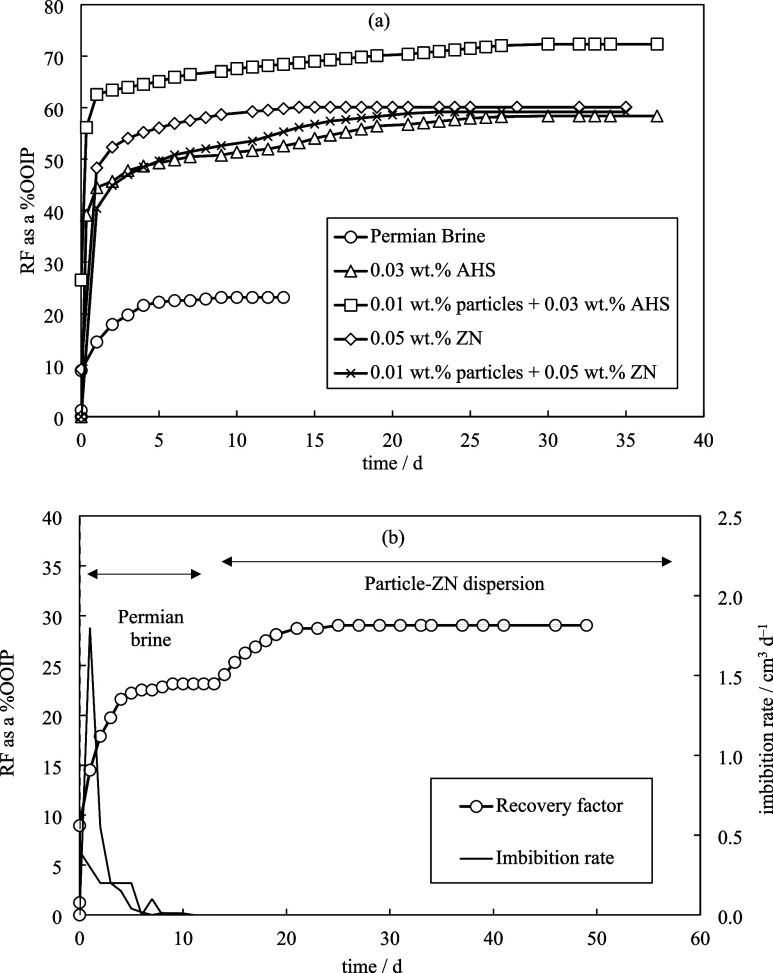
(a) Oil recovery factor (RF) for the crude oil-saturated cores imbibed with different solutions and dispersions in secondary mode at 75 °C. All solutions/dispersions were used in Permian brine. (b) Imbibition of crude oil-saturated core with Permian brine (secondary mode) and dispersion of 0.01 wt % ES-coated silica and 0.05 wt % ZN in Permian brine (tertiary mode) at 75 °C. The standard deviations of the oil recovery factor and imbibition rate are below 0.5% and 0.2 cm^3^ day^–1^, respectively.

Spontaneous imbibition tests were conducted to assess the effectiveness of formulations in enhancing oil recovery. Given the consistently low measurements of all oil–water interfacial tension (see Section 3.4), the blends exhibiting the minimum contact angles were chosen for the spontaneous imbibition tests. Figure 13 shows the secondary and tertiary oil recovery by different dispersions and solutions at 75 °C. Permian brine produced only 23% OOIP leaving a significant volume of oil within due to the rock oil-wetness and high oil–water interfacial tensions. Surfactant solutions retrieved 35–37% more oil compared to Permian brine. The contact angle measurements of fresh crude oil drops on the halved cores, conducted after imbibition tests, indicated a low contact angle by both surfactant solutions (θ_wo_ = 41 ± 2°). Additionally, both solutions reduced the oil–water interfacial tension to the same level thereby contributing to enhanced oil mobility.^37^ Both surfactants are thought to have induced the formation of oil-in-water emulsions during the imbibition tests, a conclusion supported by visual observations made during production readings and separate emulsion experiments (Table S6 and Figure S13). Emulsions have a higher viscosity than water leading to enhanced oil mobility. Based on the interfacial tension and emulsion results, neither of the surfactants exhibited a three-phase system associated with ultralow interfacial tensions.

The ultimate oil recovery factor of the AHS solution was enhanced by ∼14% OOIP when 0.01 wt % particles were added. Similarly, small post-imbibition oil–water contact angles on cores and oil–water interfacial tensions were observed when the AHS surfactant solution or surfactant-particle dispersion was used. However, the addition of ES-coated silica to AHS solutions was observed to create more stable oil-in-water emulsions (Figure S13) which subsequently produced more residual oil. On the other hand, ZN surfactant and ZN+ particles both resulted in a similar oil recovery factor. The oil production by the ZN solution stopped after 2 weeks but a more continuous oil production was observed in the presence of particles. The postimbibition contact angles and the oil–water interfacial tensions were similarly low with or without particles in ZN solutions. Therefore, it is thought that the inefficiency of emulsification is the reason for the lack of improved oil recovery with the blend of ZN and particles. The mixture of particles and AHS outperformed that of ZN in terms of ultimate oil recovery.

Replacement of Permian brine with particle-ZN dispersions could enhance oil recovery by 6% OOIP in tertiary mode (Figure 13b). This additional oil recovered by the blend is the residual oil previously trapped due to the high capillary pressures available in the pore throats of the rock which could not be produced by brine alone. It is concluded that EOR by dispersions in the secondary stage is more efficient than in the tertiary stage.

## 4 Conclusions

In this study, aqueous nanoparticle-surfactant mixtures were investigated in a formation brine for EOR in calcite-rich reservoirs. The primary innovation of this research is the stabilization of silica nanoparticles in formation brine using a silane, achieving long-term stability at high temperatures (75 °C) for over 7 months. These stabilized particles were then combined with two promising commercial surfactants to enhance their effectiveness for EOR. The study focused on using low particle concentrations to facilitate their field applications. The properties of silinized particles with and without surfactants were determined and their effects on reservoir properties were studied to determine effective formulations for spontaneous imbibition tests. The key findings are as follows:

- An optimum silane/silica ratio was obtained for ES-coated silica above which excess silane leads to particle aggregation. Surfactants were shown to further sterically stabilize silanized particles in DIW through physical adsorption but they are probably ineffective in brine. Therefore, careful attention must be given to bare nanoparticles as surfactant carriers since surfactant desorption from particle surfaces by ions may cause particle aggregation and formation damage. Electrostatic stabilization through pH adjustment was found to increase colloidal stability significantly. The particles were also found to be stable when flowing in porous media.
- The results on oil–water contact angle, surfactant adsorption onto rock and SEM-EDS were largely consistent. The particle-zwitterionic surfactant mixture showed a pH-responsive behavior in which particles might serve as carriers (zwitterionic pH) or surface activity improvers (high pH) for surfactant. The former provided the largest interactions of zwitterionic surfactant with moderately anionic particles (through V-shaped adsorption) and crude oil components (through ion pairs) leading to a synergistic contact angle reduction. The latter led to excess surfactant adsorption on rock resulting in a high oil–water contact angle.
- ZN had a narrower zero net charge pH range compared to AHS. Oil–water interfacial tensions decreased considerably on the addition of both zwitterionic and binary zwitterionic-nonionic surfactants to DIW or Permian brine at ∼CMC with and without particles (more so for ZN). Temperature elevation increased the crude oil–water interfacial tension in the presence of surfactants (with and without particles) probably due to reduced solvation of surfactant molecules at the interface by counterions (more so for ZN). Emulsions of AHS were found to be more stable than those of ZN (with or without particles).
- Surfactants considerably increased the oil recovery of brine in secondary mode due to their involvement in lowering the oil–water interfacial tension and oil-wetness of rock. The ultimate oil recovery factor with zwitterionic AHS was enhanced significantly on adding only 0.01 wt % ES-coated silica while no improvement was observed in that of ZN. This was linked to the emulsification power of blends. Additional oil recovery was also observed in tertiary mode by the blend of particles and surfactant implying the efficiency of the formulation in recovering residual oil. In flowing mode, differential pressure fluctuations decreased as follows: particle dispersion > surfactant-particle dispersion > surfactant solution > Permian brine, serving as evidence that particles can drastically cause temporary permeability reductions to reroute the injected phase to nearby pore throats for higher EOR.

Finally, it is worth mentioning that while this work has shown encouraging outcomes, additional investigation is required to examine the possible disadvantages of these mixtures for EOR, particularly their complex interactions with reservoir fluids which may affect their stability and performance. Understanding these interactions is essential for optimizing formulation design and deployment in practical EOR applications. Additionally, it is recommended that future research extend this formulation study to other reservoir types such as sandstones.

## Supporting Information Available

Literature review and specific experimental details on materials (rock, surfactants and synthesized nanoparticles); experimental results on rock wettability alteration; surfactant adsorption onto rock and emulsions (PDF)
