# Supplementary material for: Surfactant-Nanoparticle Formulations for Enhanced Oil Recovery in Calcite-Rich Rocks
Source: Langmuir. 2024 Nov 12;40(47):24989–5002. doi: 10.1021/acs.langmuir.4c03100 (PMC11603775; doi:10.1021/acs.langmuir.4c03100)
Supplement: Supplementary file 1 — la4c03100_si_001.pdf [file la4c03100_si_001.pdf]

Electronic Supplementary Information (ESI)

Surfactant-Nanoparticle Formulations for Enhanced Oil Recovery in Calcite-rich Rocks

Hosein Rezvani,[a] Bernard P. Binks[a,*] and Duy Nguyen[b]

[a] *Department of Chemistry, University of Hull, Hull. HU6 7RX. UK*

[b] *ChampionX, 11177 S. Stadium Drive, Sugar Land, Texas 77478, USA*

* Corresponding author: b.p.binks@hull.ac.uk

This supplementary document serves as an extension to the original manuscript offering additional details and presenting further results to enhance the comprehensive understanding of the research findings.

1. Literature review

Table S1 compares the EOR performance of various surfactant types and highlights the stability advantages of zwitterionic and nonionic surfactants.

Table S1. Comparison of different surfactants for EOR.[1]

Property	Zwitterionic	Nonionic	Anionic	Cationic
Headgroup Charge	Both + and – (net neutral)	No charge	–	+
pH Stability	Stable over a wide pH range		Limited by pH<br>Anionics: unstable in acidic<br>Cationics: unstable in basic	
Ionic Strength	Stable in high ionic strength environments	Less affected by ionic strength	Precipitate in high ionic strength by multivalent cations and anions.	
Interfacial Tension Reduction	Effective		Highly effective	Moderate
Adsorption on Rock	Low		High	
Foaming	Moderate	Low to moderate	High	Moderate to high
Thermal Stability	High		Variable	
Reservoir Type	Broad range of reservoir conditions		Sandstone reservoirs	Carbonate reservoirs

2. Rock

Rietveld refinement on X-ray diffraction (XRD) of rock showed 97.2% calcite and 2.8% quartz in the rock (Fig. S1). The zero point charge (ZPC) of rock in 0.01 M NaCl and Permian brine was measured to be ~ 9.3 (near that of calcite at ~ 9)[2] and ~ 8, respectively using the pH drift method[3] (Fig. S1). Nitrogen Brunauer-Emmett-Teller (BET) analysis on the rock at 77 K using a Micromeritics TriStar Porosimeter showed a large BET surface area (2.9 ± 0.1 m^2 g^{-1}) implying that the rock has a high potential for adsorbing chemicals in EOR.

3. Surfactants

3.1. Thermal tolerance

The thermal tolerance of surfactants was analyzed by thermogravimetric analysis (TGA) on surfactants standing at 100 °C after 30 days (Fig. S2). The results show a small weight loss up to 150 °C (due to the water removal) followed by a more significant weight loss due to the oxidation of hydrocarbon groups in the tails of both surfactants. When the organic molecules react with oxygen at high temperatures, oxides such as CO_2, H_2O and CO are produced which is referred to as combustion or combustion-like oxidation. Overall, excellent thermal stability up to 150 °C (less than typical reservoir temperature) is shown with both surfactants for EOR operations.

Fig. S2 also shows the appearance of surfactant solutions at different temperatures. The surfactants did not show any precipitation in Permian brine after a month at 100 °C.

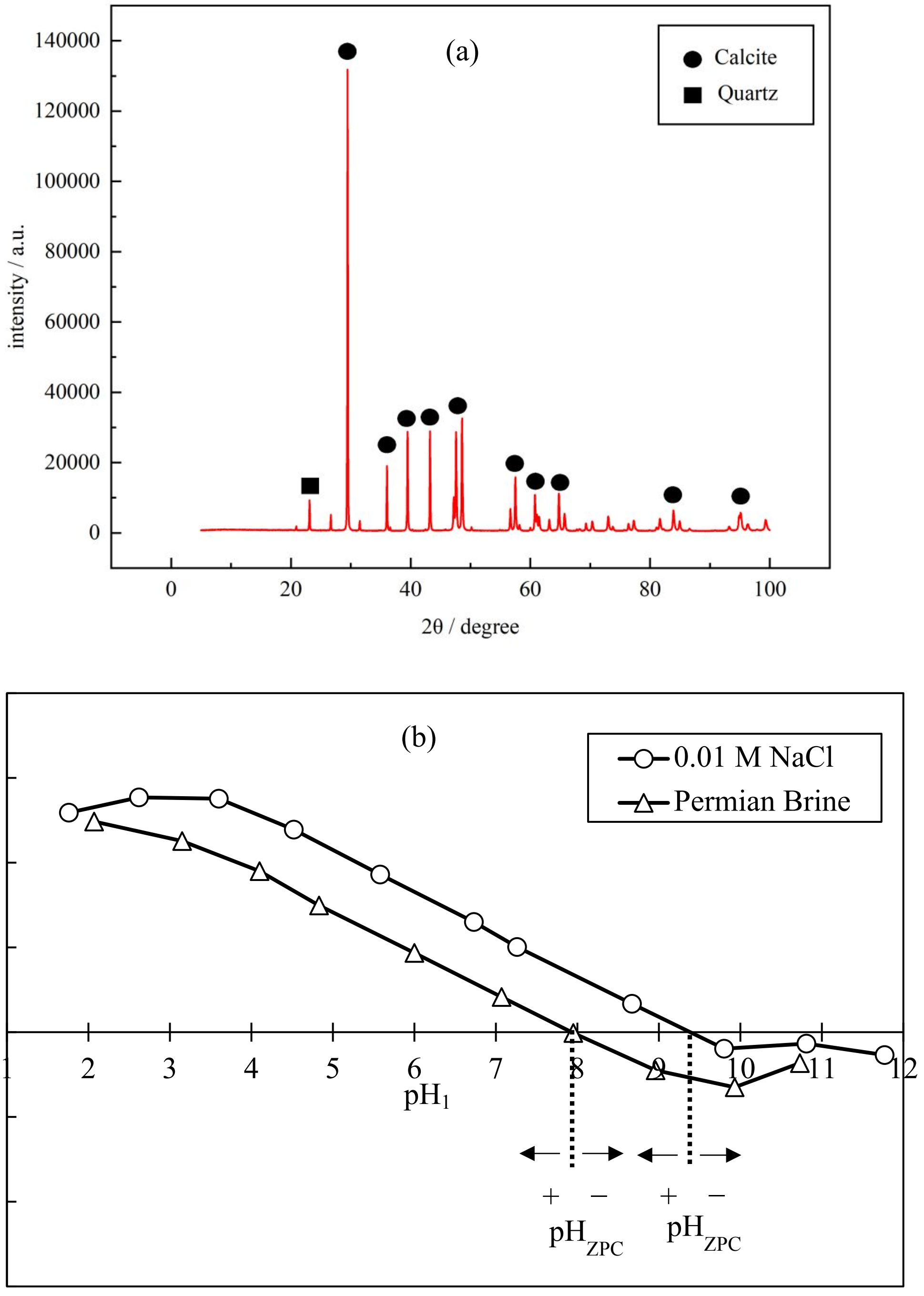

Fig. S1. (a) XRD of rock, (b) pH variations on equilibration of rock powder (10 μm) with 0.01 M NaCl or Permian brine for the determination of ZPC. pH_1 and pH_2 represent the initial and equilibrium (after 48 h) pH of rock powder suspensions, respectively.

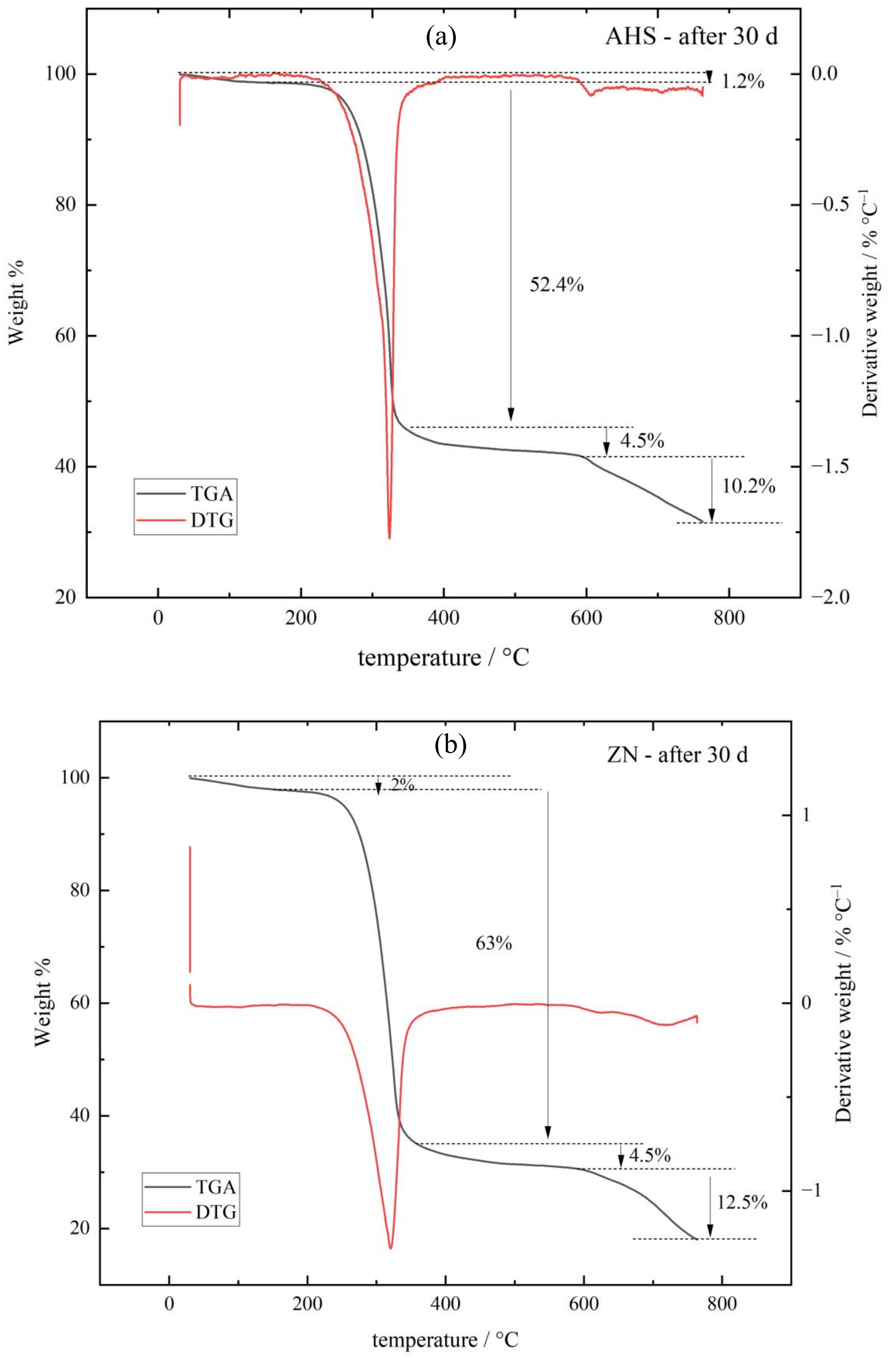
(a)
AHS - after 30 d
1.2%
52.4%
4.5%
10.2%
TGA
DTG
Weight %
Derivative weight / % °C⁻¹
temperature / °C
0
200
400
600
800
100
80
60
40
20
0.0
−0.5
−1.0
−1.5
−2.0
(b)
ZN - after 30 d
2%
63%
4.5%
12.5%
TGA
DTG
Weight %
Derivative weight / % °C⁻¹
temperature / °C
0
200
400
600
800
100
80
60
40
20
1
0
−1

(c)

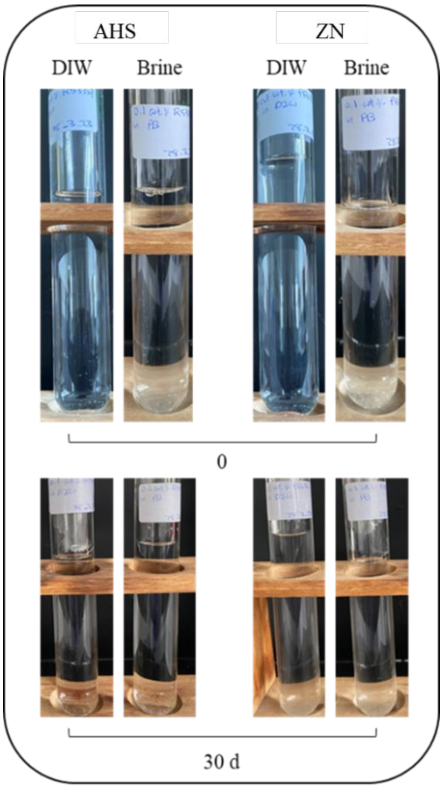

Fig. S2. TGA and differential thermogravimetry (DTG) of (a) AHS and (b) ZN surfactant after standing at 100 °C for 30 days. (c) Appearance of 0.1 wt.% AHS or ZN in DIW and Permian brine at 20°C and 100 °C at different times.

3.2. Micelle charge

Fig. S3 shows the charge behaviour of surfactants as a function of pH. The AHS surfactant showed a net zero charge at a pH of 5.5 – 8.0 at 25 °C while this region was narrower for the ZN surfactant (pH = 7.5 – 8.4). Below this range, both surfactants show a more positive charge because of the protonation of the sulfonate group which leaves the cationic quaternary ammonium determining the headgroup charge. At high pH, due to the deprotonation of the sulfonate group, the negative charge of the surfactant headgroup becomes dominant which renders the surfactant weakly anionic. In addition, ZN has relatively lower zeta potential values due to the presence of nonionic surfactant monomers in alkyl hydroxysultaine micelles.

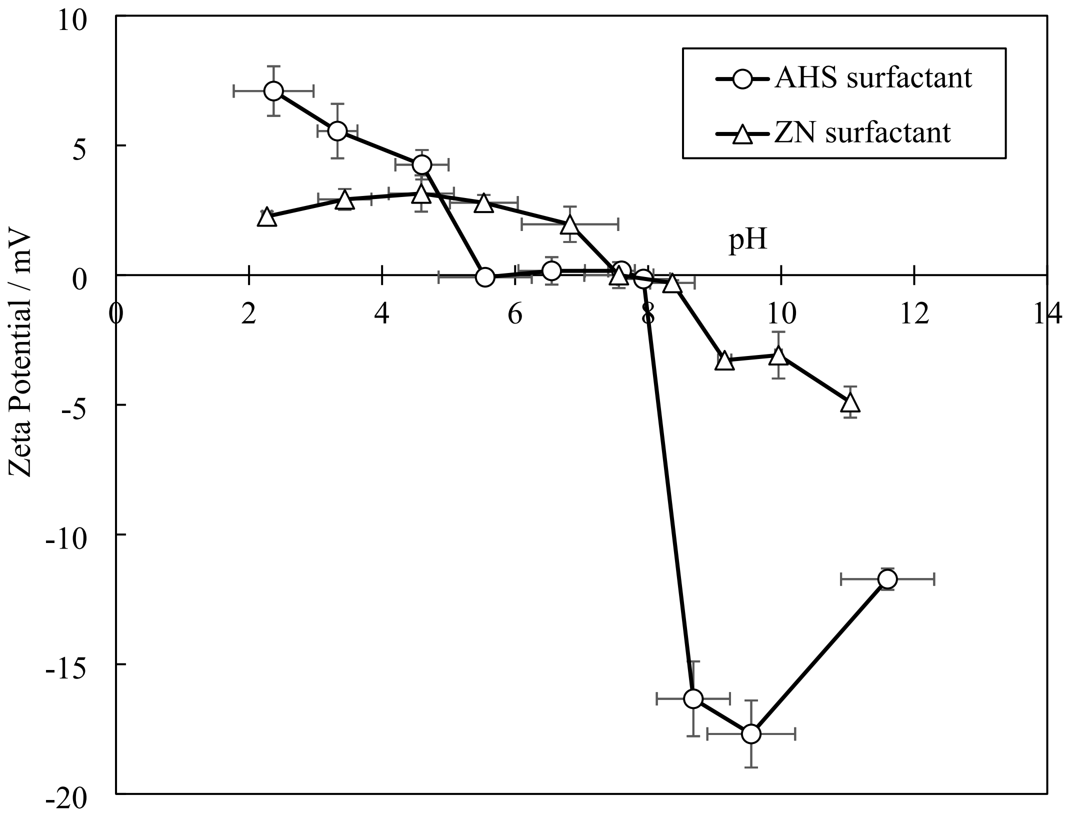

Fig. S3. Zeta potentials of AHS and ZN surfactants in DIW at different pH values at 25 ± 0.1 °C. [surfactant] = 0.1 wt.%.

4. ES-coated silica

4.1. Surface chemistry

Fig. S4 shows the XRD and TGA of ES-coated silica. ES-coated silica has additional XRD peaks at 2θ = 43° and 50° which are related to the grafting of silica with silane by covalent bonds. No further peaks in the pattern prove the absence of impurities in the sample. The silane coverage on silica can be calculated using TGA:[4]

$$\phi_i = \frac{f_{org}}{\left(1 - f_{org}\right) \times SSA \times MW} \times 10^6 \qquad \text{(S1)}$$

where ϕ_i is the organic coverage in μmol m^{-2} of the particle surface, f_{org} is the weight loss fraction due to the loss of particle surface organic molecules, *SSA* is the specific surface area of bare silica (331 m^2 g^{-1}) and *MW* is the molecular weight of the silane in g mol^{-1} that can be removed by TGA. The ES coverage on silica was calculated at 55% of a full monolayer.

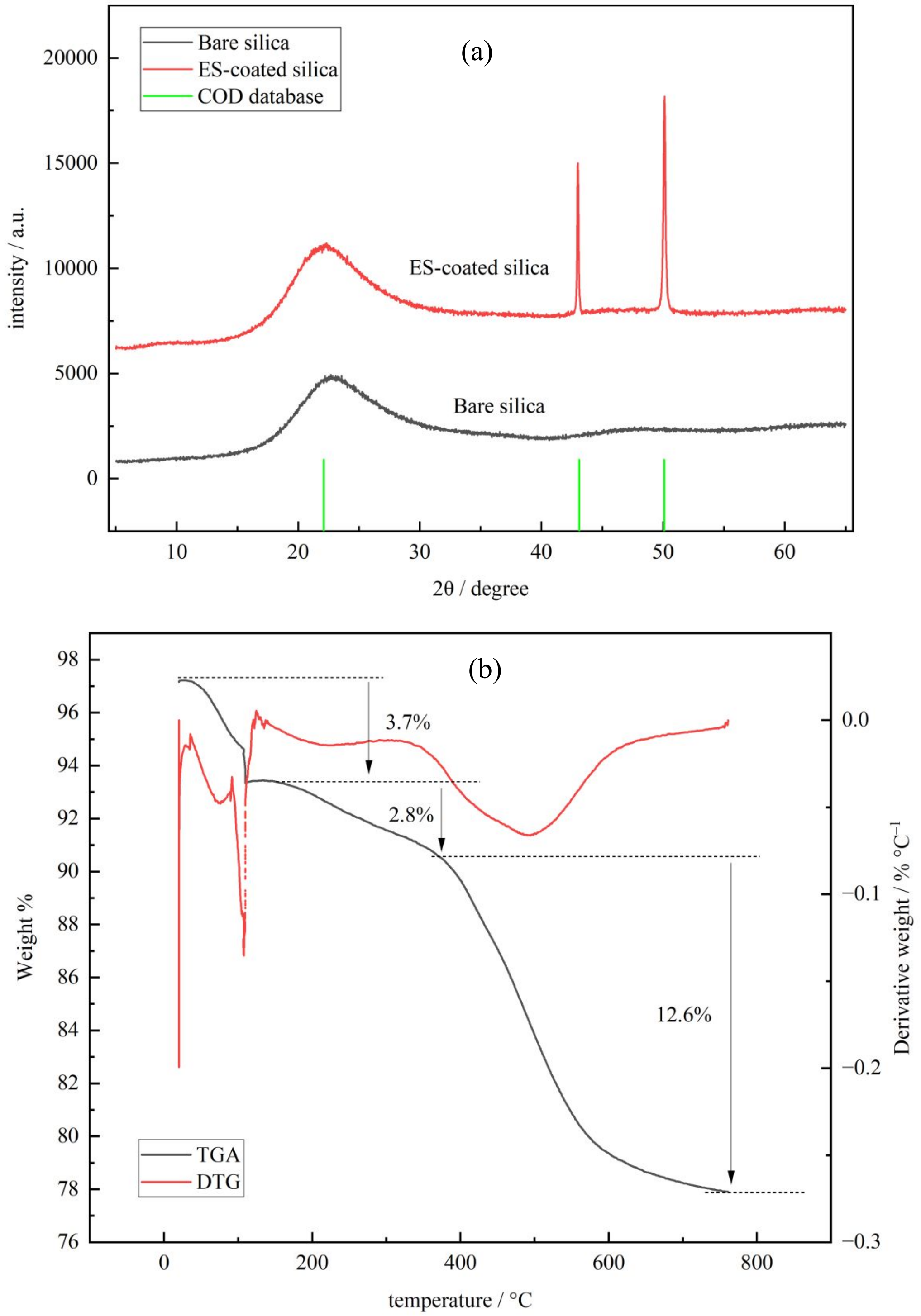

Fig. S4. (a) XRD and (b) TGA-DTG of ES-coated silica.

4.2. Particle surface activity

Air-water surface tension measurements show that particles are weakly surface-active (Table S2) due to their surface hydroxyl groups on both uncoated and coated sites. However, the

hydrocarbon group in the silane tip can induce partial hydrophobicity.[5] High air-water surface tension measurements in Table S2 show that particles are weakly surface-active.

Table S2. Air-water surface tensions of different concentrations of ES-coated silica in DIW and Permian brine at original and reduced pH (by HCl) at 25 °C.

[particle]/wt.%	γ_{aw}/mN m^{-1}			
	**DIW**		**Permian brine**	
	**pH = 7 – 9**	**pH = 4**	**pH = 6.2 – 7.5**	**pH = 4**
0	72.0 ± 0.1	72.0 ± 0.1	74.3 ± 0.2	74.3 ± 0.2
0.01	65.6 ± 0.3	65.4 ± 0.4	74.4 ± 0.1	67.2 ± 0.4
0.03	64.8 ± 1.8	66.2 ± 0.8	70.7 ± 0.5	67.7 ± 0.3
0.05	68.1 ± 0.3	64.0 ± 0.5	70.1 ± 0.9	67.1 ± 0.7
0.07	64.9 ± 0.7	61.5 ± 1.2	68.0 ± 0.8	70.7 ± 1
0.10	66.0 ± 1.4	61.1 ± 0.6	65.8 ± 1	66.8 ± 0.6

4.3. Particle stability

Fig. S5 shows the stability of 0.1 wt.% ES-coated silica in DIW and Permian brine at original pH and reduced pH. The screening of particle surface charges by cations decreases significantly with pH reduction and as a result the dispersions are rendered stable for a long time. Table S3 shows the initial particle diameter and zeta potential of bare and ES-coated silica. pH reduction was also found to be efficient in increasing the re-dispersibility of particles after initial sedimentation. The initial particle diameter of all dispersions in DIW and Permian brine at the original and reduced pH was 22 ± 3 nm. HCl did not affect the initial particle diameter but stabilised the particles long-term in brine at high temperatures. The appearance of blends of ES-coated silica and surfactants at two temperatures with time is presented in Fig. S6.

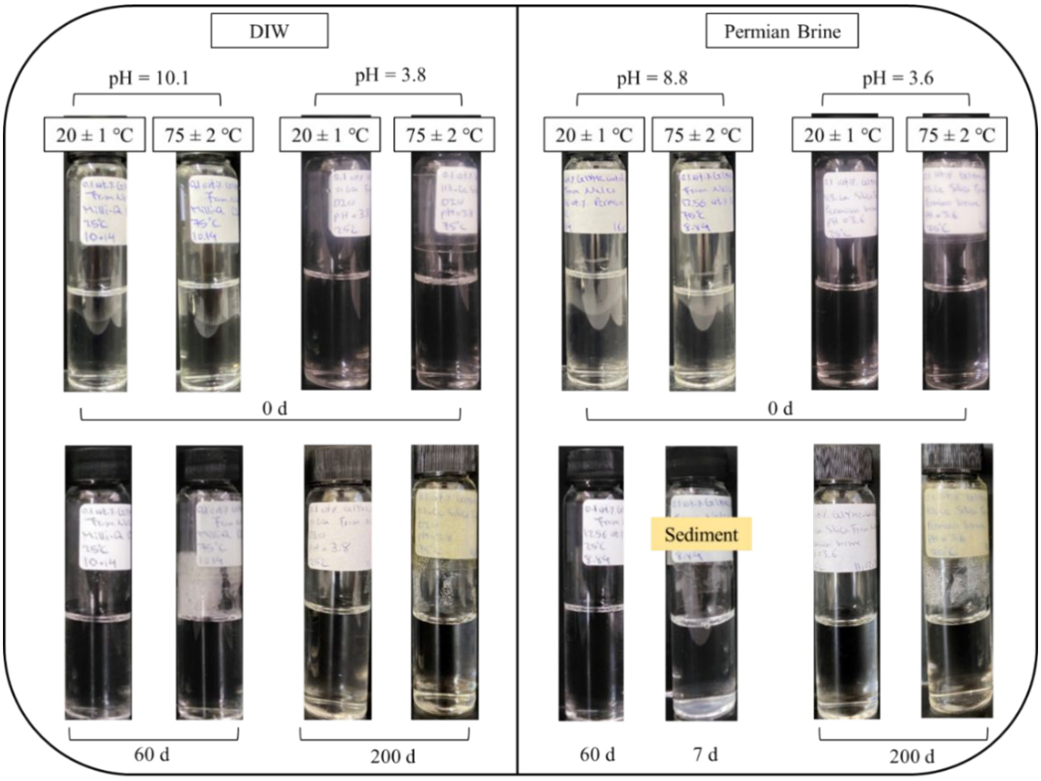

Fig. S5. Appearance of dispersions containing 0.1 wt.% ES-coated silica in DIW and Permian brine at the original and reduced pH (by HCl) at two temperatures at different times.

Table S3. Effect of silane coverage and particle concentration on the initial particle diameter and zeta potential of bare and ES-coated silica in DIW at 25 °C.

[particle]/wt.%	Bare silica (pH = 8.5 – 10)		ES-coated silica (55% coverage) (pH = 7 – 9)	
	d/nm	ζ/mV	d/nm	ζ/mV
0.01	18 ± 0.1	– 41.5 ± 0.5	22 ± 0.1	– 18.6 ± 3.1
0.03	17 ± 0.4	– 42.3 ± 0.3	21 ± 0.2	– 21.8 ± 1.9
0.05	16 ± 0.2	– 40.7 ± 0.6	21 ± 0.1	– 24.2 ± 1.0
0.07	15 ± 0.3	– 42.0 ± 0.5	20 ± 0.2	– 26.7 ± 1.1
0.1	15 ± 0.5	– 43.8 ± 1.2	20 ± 0.5	– 26.9 ± 0.6

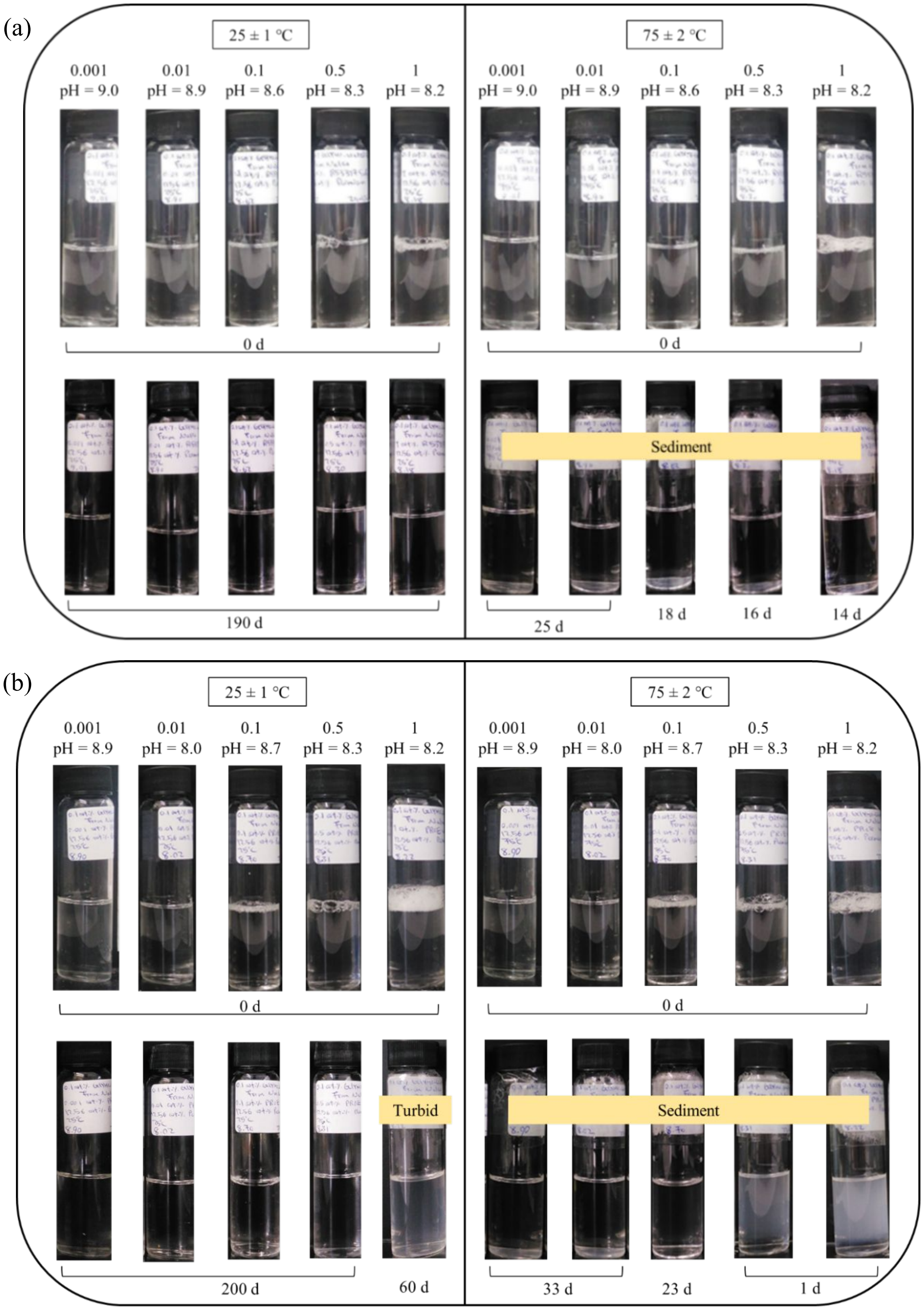

Fig. S6. Appearance of dispersions containing 0.1 wt.% ES-coated silica in a blend with different concentrations of (a) AHS and (b) ZN in Permian brine at original pH at two temperatures.

The pressure profiles created by injecting Permian brine followed by ZN solutions with and without 0.01 wt.% ES-coated silica particles at 75 °C are shown in Fig. S7. The same pressure profile was observed for the AHS surfactant.

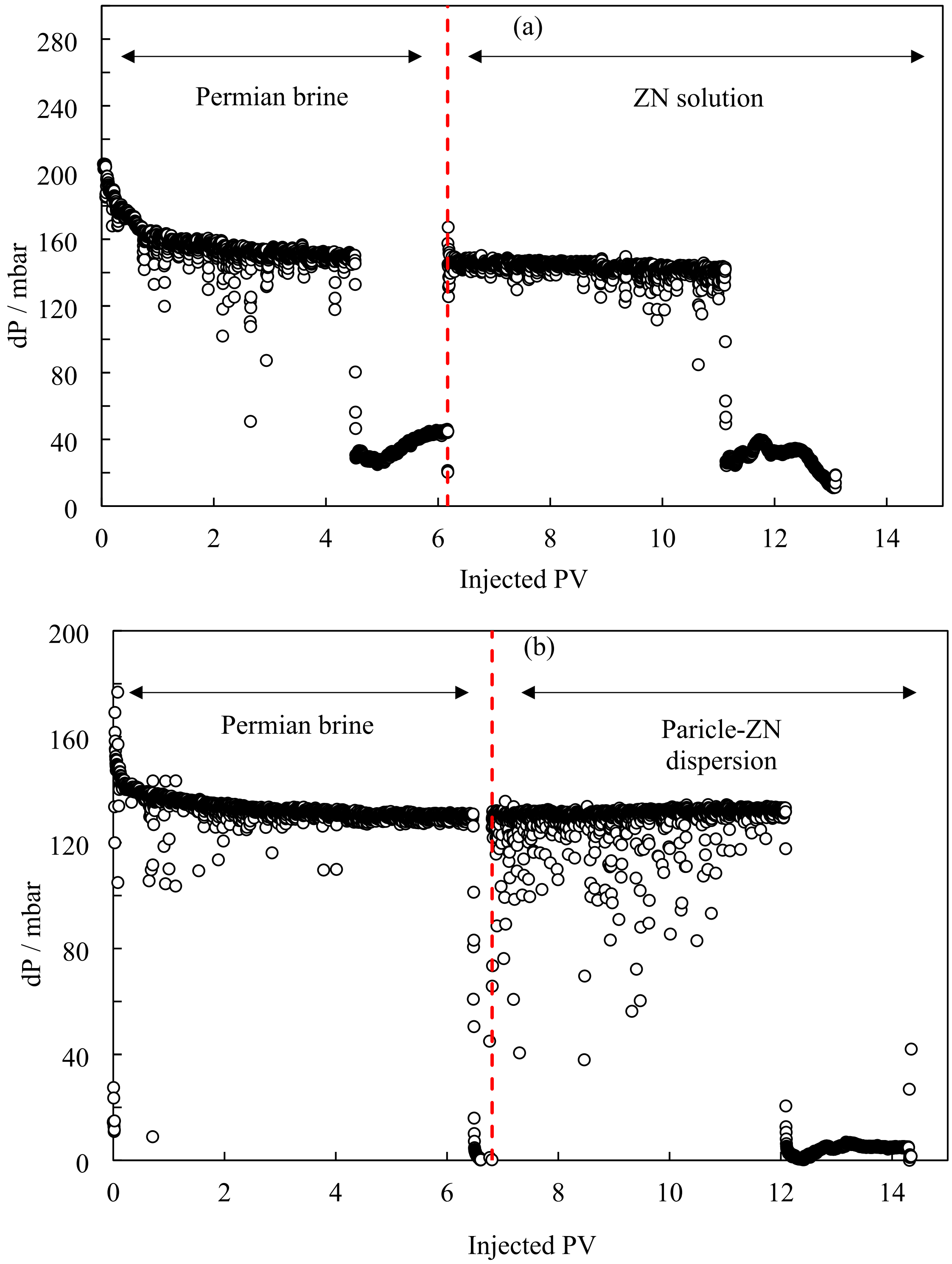

Fig. S7. Differential pressure *versus* injected pore volume for a dried core injected with Permian brine followed by 0.05 wt.% ZN in Permian brine (a) without or (b) with 0.01 wt.% ES-coated silica at 0.1 cm^3 min^{-1} and 75 °C.

5. Contact angle measurement

5.1. Particles

Table S4 shows the oil-water contact angle for bare and ES-coated silica, respectively. Once the disjoining pressure caused by particle layering between the oil drop and the solid surface exceeds the adhesion force between the oil drop and the rock surface, the oil drop starts to detach leaving the surface more water-wet.[6] As ES is nonionic, no electrostatic interactions between the ligand itself and the solid surface are expected. The negative charge of ungrafted sites on silica however can have a share in the electrostatic adsorption of particles on the cationic rock surface which reduces the positive charge of the rock surface and leads to a reduction in the attraction between crude oil components and the rock surface. It is worth mentioning that the electrostatic attraction between the positive headgroup of zwitterionic surfactant and ES-coated silica (through V adsorption) is believed to be weaker than that of bare silica and a cationic surfactant due to the silane coating (lower reactive silanol groups) and lower cationic charge in the zwitterionic surfactant headgroup. That is why the oil-water contact angles of bare silica are significantly lower than those of ES-coated silica. Particle adsorption also increases the hydrophilicity of the rock surface due to increased water molecules. The particles can also have electrostatic interactions with cationic components of the crude oil, like nitrogen-based groups. High particle concentrations raise the pH resulting in a higher number of negative charges on particle surfaces. As a result, the adsorbed anionic particles on the rock attract the oppositely charged components of crude oil more strongly promoting both particle and rock hydrophobicity.

Table S4. Equilibrium contact angles of oil droplets on oil-conditioned rock treated with different concentrations of bare silica and ES-coated silica in DIW and Permian brine.

[particle]/wt.%	$\theta_{w,o}$/°		
	Bare silica in DIW	ES-coated silica	
		DIW	Permian brine
0	171 ± 8	171 ± 8	171 ± 8
0.01	113 ± 4	139 ± 5	143 ± 6
0.03	124 ± 7	138 ± 7	167 ± 2
0.05	49 ± 9	145 ± 6	160 ± 3
0.07	38 ± 7	165 ± 2	167 ± 2
0.10	86 ± 8	162 ± 3	165 ± 2

5.2. Surfactant-particle mixtures

Fig. S8 shows the oil-water contact angle and pH measurements of blends of AHS or ZN and ES-coated silica in DIW. The minimum contact angle here is 48° which is observed with the blend containing 0.01 wt.% particles and 0.03 wt.% AHS in DIW. This minimum contact angle is lower than that of particles alone or surfactant alone in DIW indicating a synergy by blending the chemicals probably due to the mixed monolayer of particles and surfactant formed on the rock. The change in pH with particle or surfactant concentration affects the charge of the zwitterionic AHS and particles. High concentrations of particles and surfactant increase the pH towards the upper limit of the isoelectric region which may create repulsion between anionic particles and weakly anionic surfactant molecules, making surfactant molecules adsorb more on the rock surface, consistent with increased AHS adsorption onto the rock at high particle and surfactant concentrations.

Fig. S8 also shows the oil-water contact angle and pH measurements for blends of ZN and ES-coated silica in DIW. Compared to the contact angle by 0.07 wt.% ZN in DIW, a further 61° reduction in contact angle is observed upon the addition of 0.01 wt.% particles. At high particle loadings (0.1 wt.%), the surfactant mostly adsorbs onto the particle surface which makes the blend less effective for rock wettability alteration due to the surfactant depletion. This is consistent with the reduced ZN adsorption on the rock with the addition of particles in DIW.

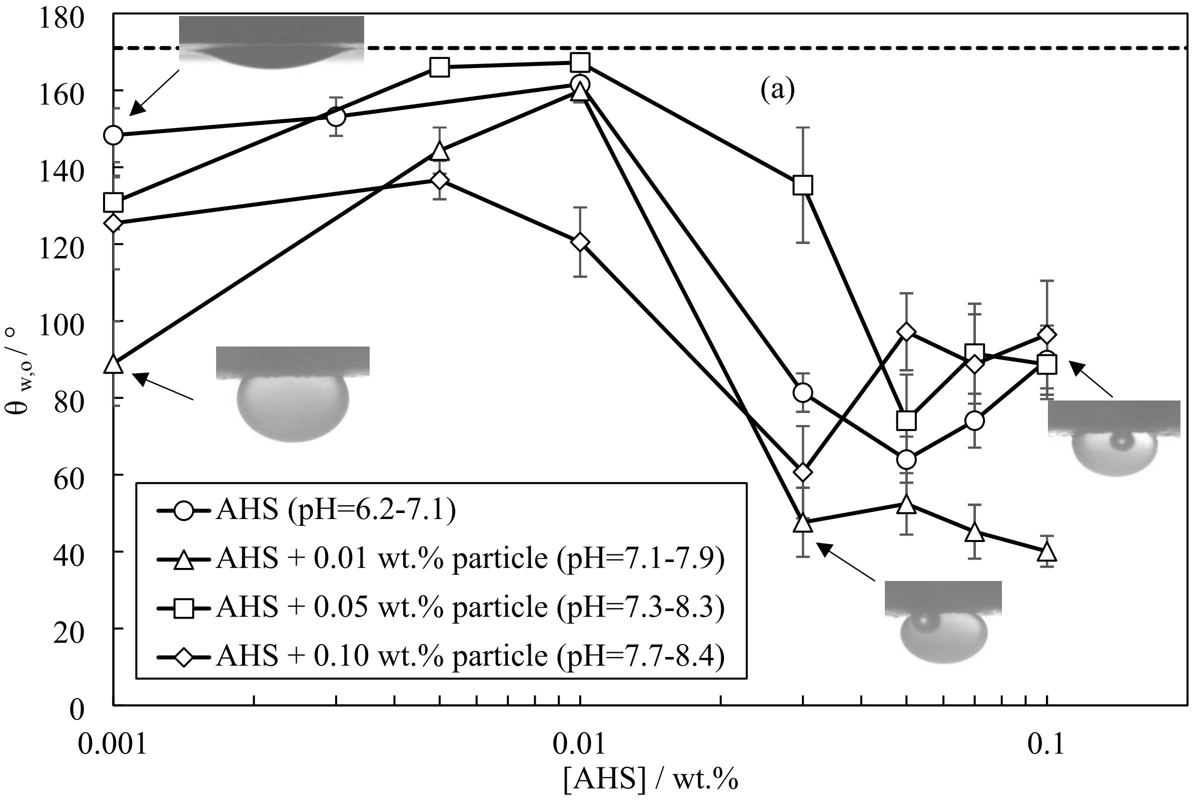

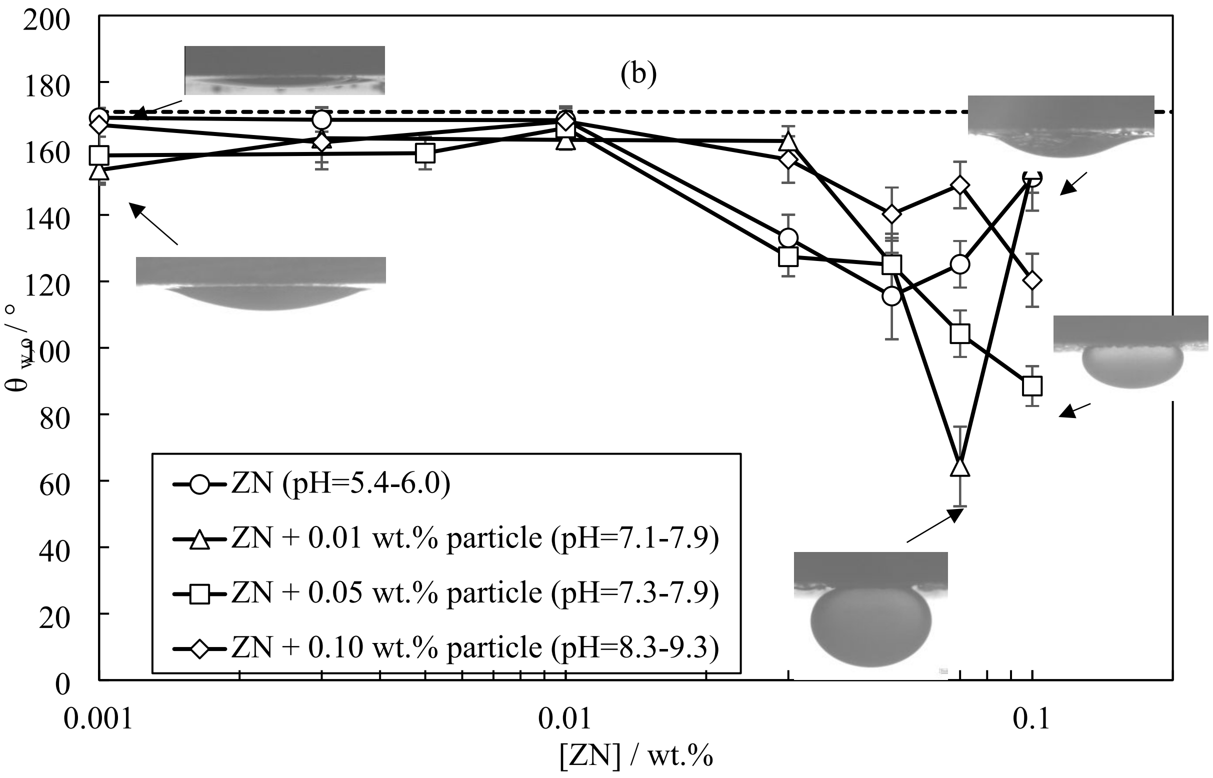

Fig. S8. Equilibrium contact angles of crude oil droplets on oil-conditioned rock treated for 24 h with different (a) AHS and (b) ZN concentrations in DIW with or without different concentrations of ES-coated silica. The black dashed line shows the contact angle of oil droplets on oil-conditioned rock treated with DIW. pH decreases with increasing surfactant concentration.

6. Surfactant adsorption onto rock

The equilibrium adsorption of AHS and ZN onto rock powder was investigated at 25 °C. The experimental data were modelled with different non-linear adsorption isotherms including Sips, Langmuir, Freundlich, Redlich-Peterson and Temkin models using the Solver add-in in Excel. The results showed that the data are best fitted with Redlich-Peterson (highest R^2). The Redlich-Peterson equation is given by:[7]

$$Q_e = \frac{K_{RP}C_e}{1 + \alpha_{RP}{C_e}^n} \tag{S2}$$

where Q_e is the adsorbed amount (mg g^{-1}), K_{RP} is the Redlich-Peterson constant (L mg^{-1}), C_e is the equilibrium surfactant concentration, n is an exponent and α_{RP} is the Redlich-Peterson constant (L mg^{-1})^n. The parameters of the Redlich-Peterson model are presented in Table S5.

Table S5. Parameters of the Redlich-Peterson adsorption model fitted to the experimental adsorption of AHS and ZN onto rock with or without ES-coated silica at 25 °C.

Surfactant	[particle]/wt.%	Solvent	Redlich-Peterson model parameters		
			K_{RP}/L mg^{-1}	α_{RP}/(L mg^{-1})^n	n
AHS	0	DIW	12.33	19.32	0.59
	0.01		0.019	3×10^{-7}	0.55
	0.05		0.017	7×10^{-9}	0.07
	0.10		0.03	2×10^{-5}	0.13
	0	Permian brine	0.03	0.001	0.41
	0.01		0.06	0.002	0.48
	0.05		0.009	2×10^{-5}	0.89
	0.10		0.03	2×10^{-5}	0.13
ZN	0	DIW	0.08	0.001	0.46
	0.01		0.07	0.002	0.34
	0.05		0.008	1×10^{-5}	0.83
	0.10		0.05	0.001	0.41
	0	Permian brine	1.22	5.05	0.55
	0.01		0.07	0.02	0.10
	0.05		0.20	0.008	0.45
	0.10		0.09	0.002	0.51

Figs. S9 to S12 show the adsorption of surfactants on rock, with and without ES-coated silica. The results are consistent with contact angle measurements. Minimum contact angles were mostly observed at surfactant concentrations where maximum surfactant adsorption on rock was found. For example, an increase in the minimum oil-water contact angle from 64° to 119° was observed with the addition of Permian brine to the AHS solutions consistent with reduced surfactant adsorption upon adding brine. The change in adsorption onto the rock with the addition of Permian brine to ZN solutions is not significant which agrees with the same contact angles measured with and without the salts.

The plots show a decrease in AHS adsorption onto rock upon addition of 0.01 wt.% particles in DIW while no significant change is observed in the adsorption plot at higher particle concentrations. This agrees with oil-water contact angle measurements where it was observed that particle concentrations above 0.01 wt.% in DIW do not change the contact angle considerably. The AHS concentration causing maximum adsorption is the same as the one at which a minimum oil-water contact angle was observed. The same behaviour is observed with ZN. The surfactant concentrations in the blends causing maximum adsorption (0.05 – 0.07 wt.%) are the same as the ones causing minimum oil-water contact angles. The decreasing adsorption above the maximum also corresponds to the increasing oil-water contact angle at high surfactant concentrations.

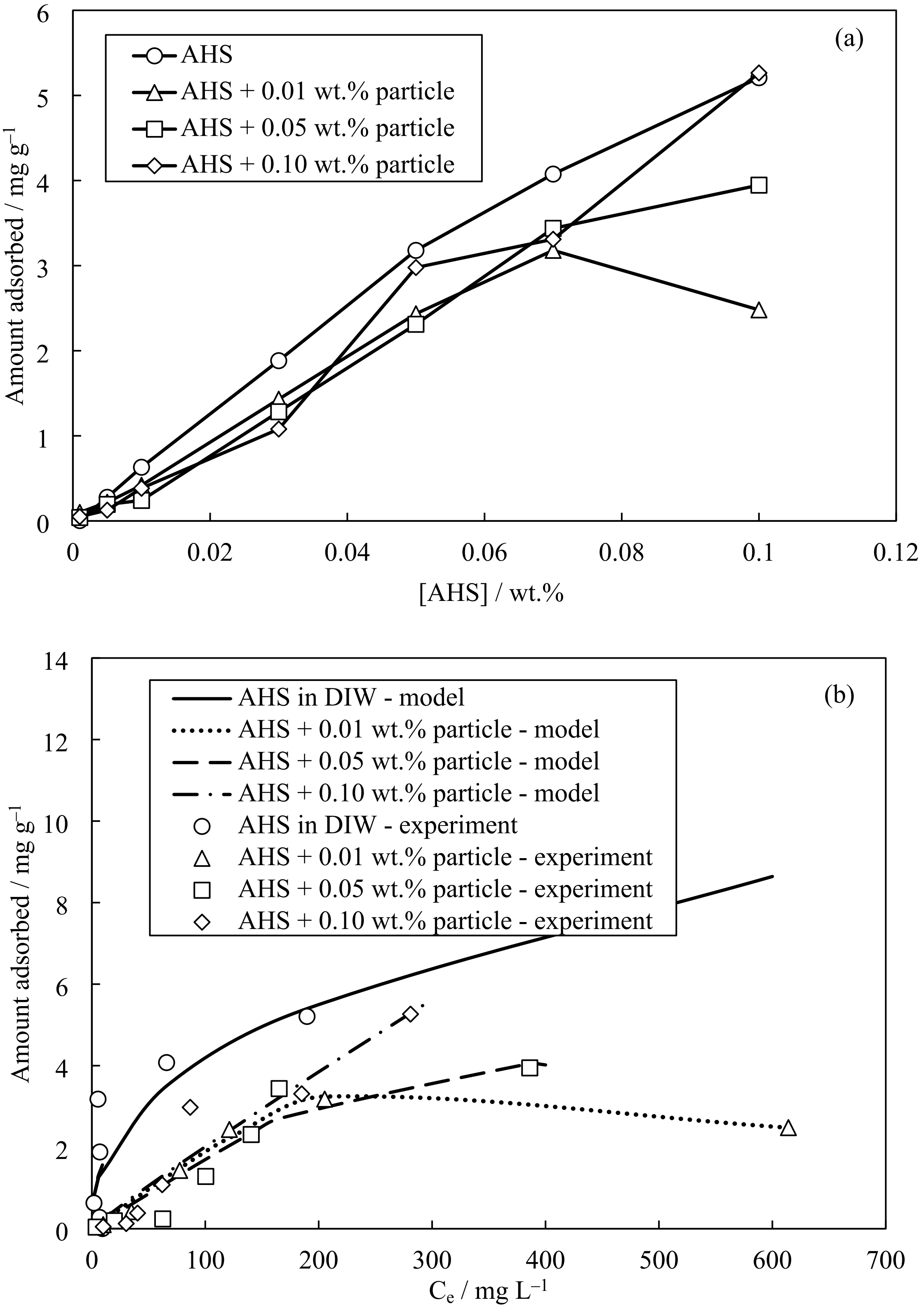

Fig. S9. Amount of AHS adsorbed onto rock at 25 °C *versus* (a) initial and (b) equilibrium surfactant concentration in DIW for different concentrations of ES-coated silica. In the lower plot, the points show the experimental data and the curves show the Redlich-Peterson adsorption isotherm.

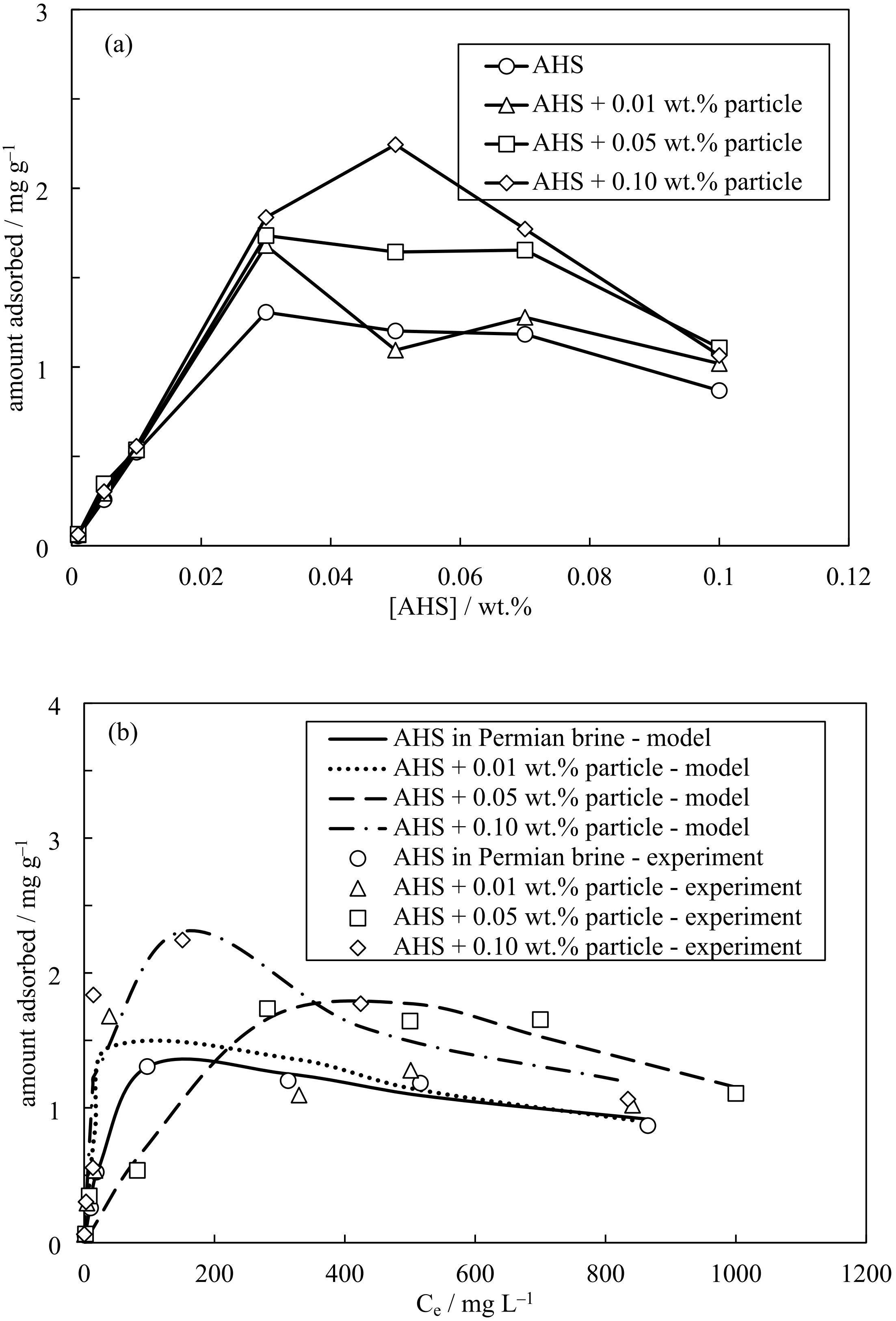

Fig. S10. Amount of AHS adsorbed onto rock at 25 °C *versus* (a) initial and (b) equilibrium surfactant concentrations in Permian brine for different concentrations of ES-coated silica. In the lower plot, the points show the experimental data and the curves show the Redlich-Peterson adsorption isotherm.

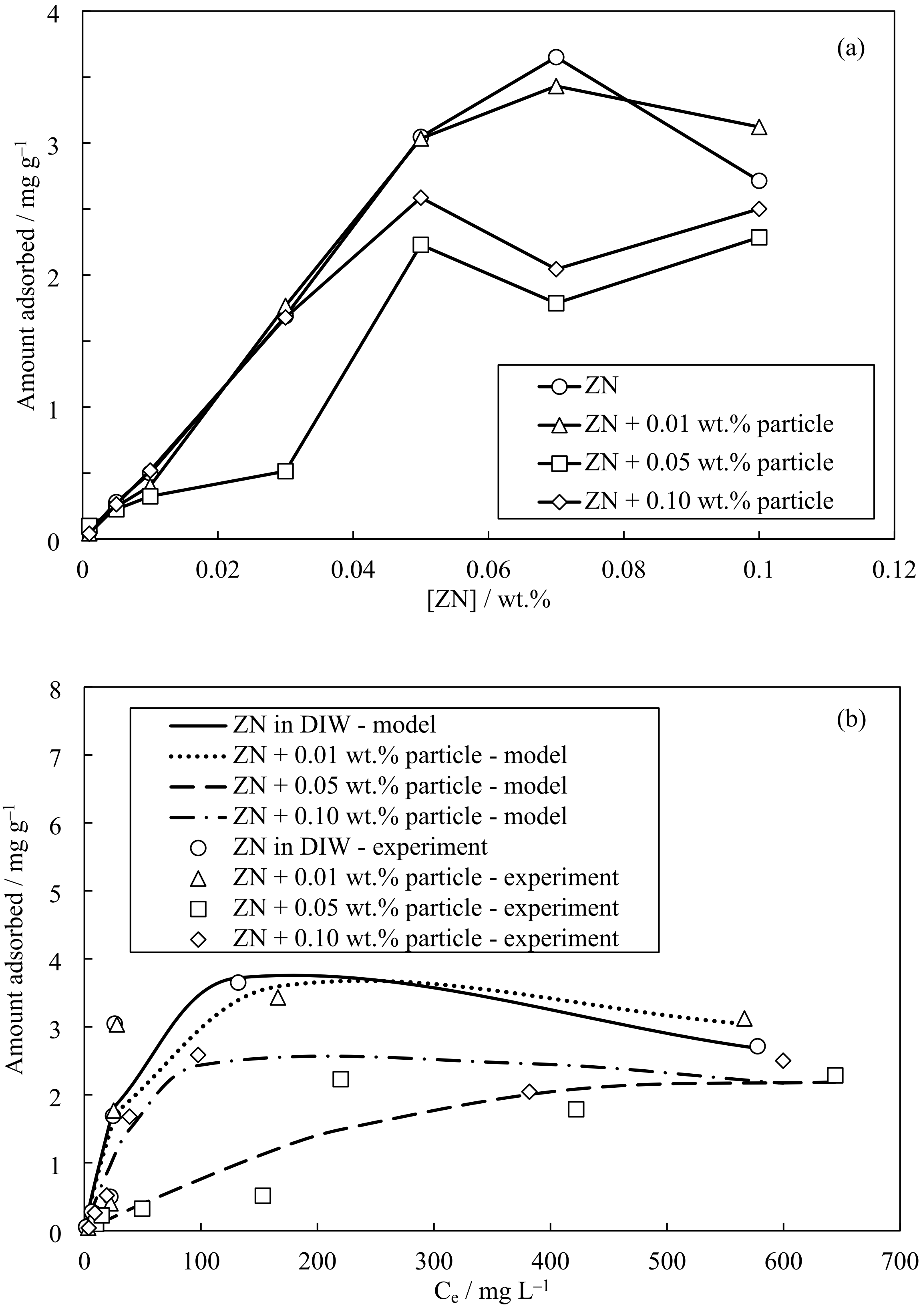

Fig. S11. Amount of ZN adsorbed onto rock at 25 °C *versus* (a) initial and (b) equilibrium surfactant concentrations in DIW for different concentrations of ES-coated silica. In the lower plot, the points show the experimental data and the curves show the Redlich-Peterson adsorption isotherm.

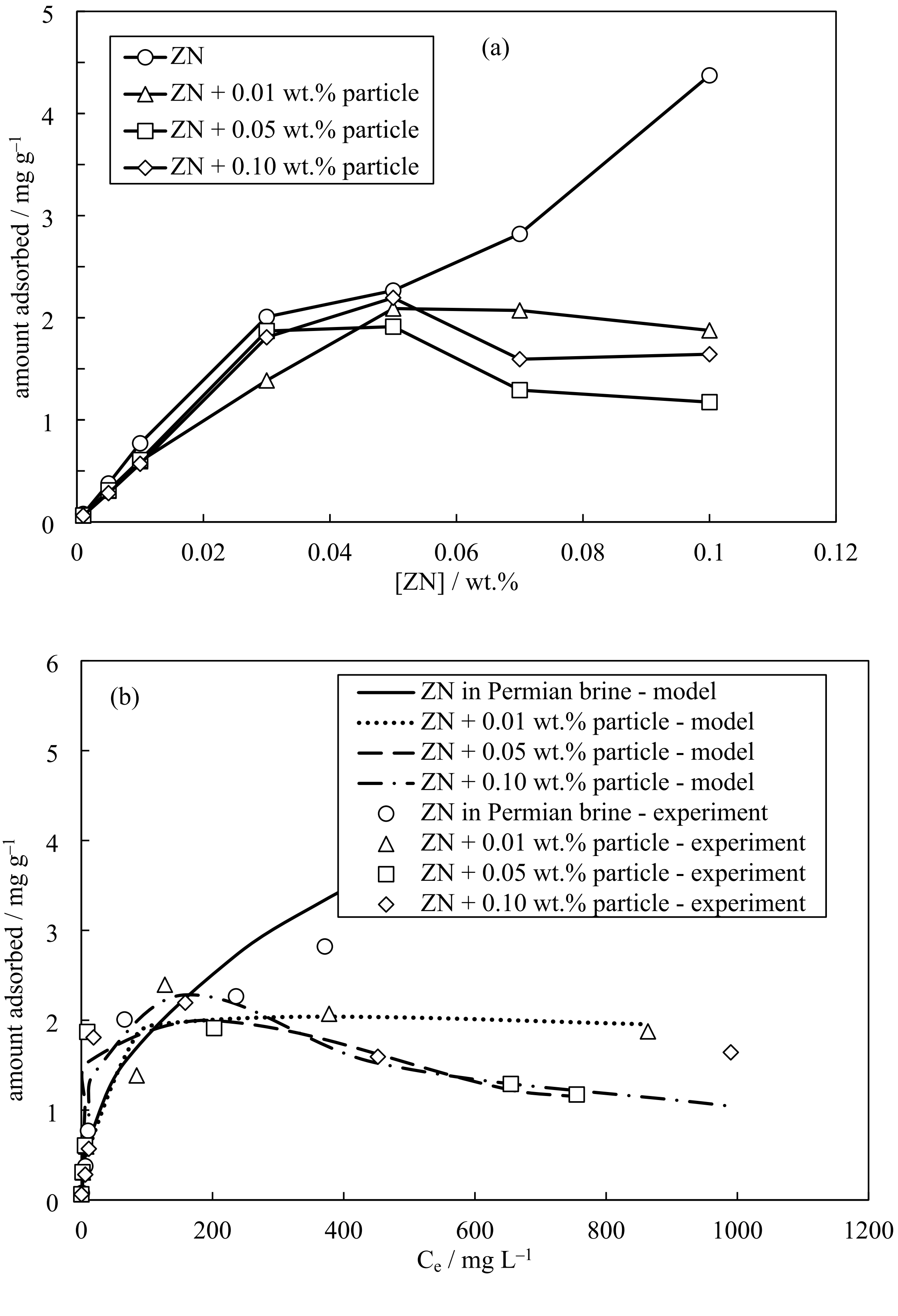

Fig. S12. Amount of ZN adsorbed onto rock at 25 °C *versus* (a) initial and (b) equilibrium surfactant concentrations in Permian brine for different concentrations of ES-coated silica. In the lower plot, the points show the experimental data and the curves show the Redlich-Peterson adsorption isotherm.

7. Emulsions

Table S6 summarises the results for emulsions made from crude oil. The emulsion of crude oil and DIW faced significant coalescence and creaming in an hour while that of Permian brine was more stable to coalescence and creaming in a month. Initially, the rapid diffusion of aqueous surfactant molecules (zwitterionic and nonionic surfactants of ZN) and crude oil surfactants (polar groups like petroleum acids) to the neat oil-water interface creates a mixed interfacial surfactant monolayer which is followed by a time-dependent movement of adsorbed crude oil surfactants to the aqueous phase to produce mixed micelles with ZN surfactant. This is evidenced by the brownish colour of the resolved water. The latter may also cause a rise in oil-water interfacial tension. When ZN is in Permian brine, the electrostatic interactions between aqueous and crude oil surfactants are reduced, resulting in a lower number of interfacial surfactant molecules and quicker phase separation. Comparing surfactants, ZN was found to be less efficient in the emulsification of oil and water (Fig. S13).

Table S6. Summary of results on oil-in-water emulsions from crude oil and different ZN solutions or bare silica and ES-coated silica in DIW and Permian brine after 30 days.

Aqueous phase	Photo	Initial microscopy	Comment
DIW	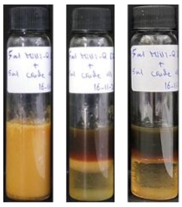 0 1 h 30 d	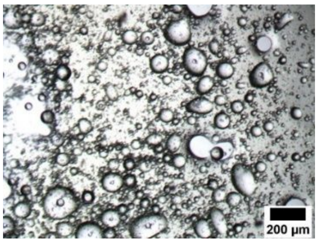 84 ± 11 µm	Mostly sodium ions interact with crude oil polar groups to stabilise the emulsions initially.
Permian brine	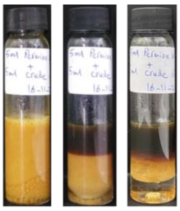 0 1 d 30 d	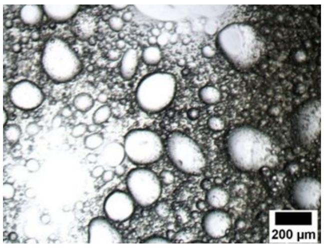 87 ± 13 µm	
0.1 wt.% ZN in DIW	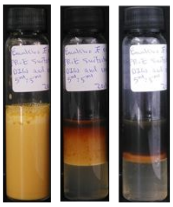 0 1 d 30 d	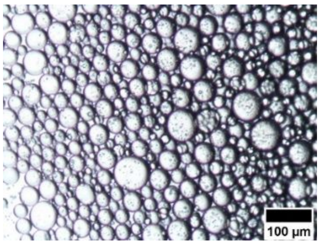 27 ± 5 µm	Permian brine made coalescence and creaming faster.<br>Brine ions lower the electrostatic interactions between aqueous and crude oil surfactants at the oil-water interface resulting in a lower number of interfacial surfactants. This is accompanied by the demulsification power of ZN.
0.1 wt.% ZN in Permian brine	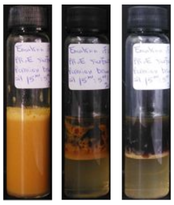 0 1 d 30 d	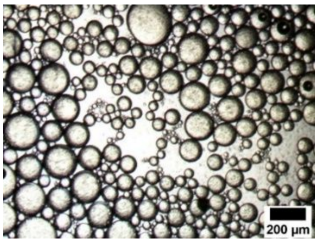 85 ± 14 µm	
0.05 wt.% bare silica in DIW	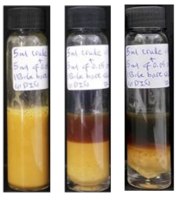 0 1 h 30 d	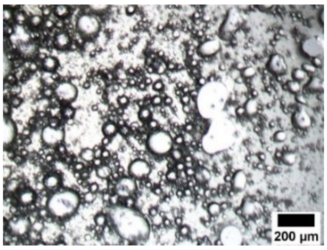 97 ± 15 µm	Particle hydrophobization extent by crude oil: bare silica > silane-coated silica.<br>Slightly aggregated bare silica particles in Permian brine can stabilise the emulsions more.

0.05 wt.% bare silica in Permian brine	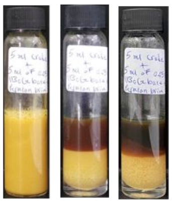 0 1 h 30 d	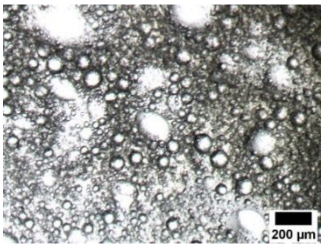 51 ± 10 µm
0.01 wt.% ES-coated silica in DIW	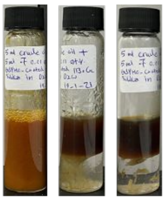 0 1 h 30 d	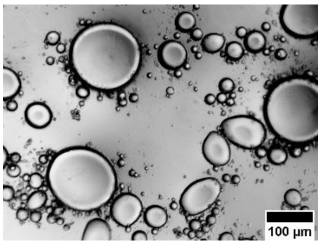 110 ± 15 µm

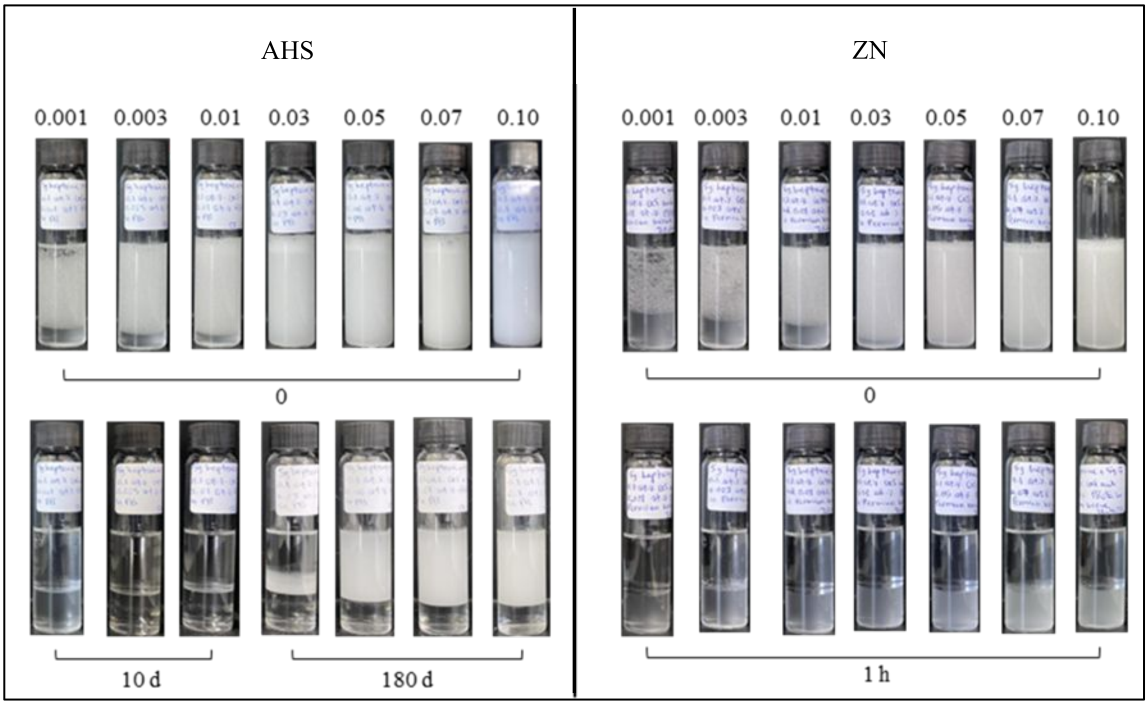

Fig. S13. Appearance of oil-in-water emulsions from 5 g heptane and 5 g of dispersions containing 0.1 wt.% ES-coated silica and different weight percentages of AHS or ZN in Permian brine.

References

(1) Isaac, O. T.; Pu, H.; Oni, B. A.; Samson, F. A. Surfactants employed in conventional and unconventional reservoirs for enhanced oil recovery—A review. *Energy Rep.* **2022**, *8*, 2806-2830. DOI: https://doi.org/10.1016/j.egyr.2022.01.187.

(2) Somasundaran, P.; Agar, G. The zero point of charge of calcite. *J. Colloid Interface Sci.* **1967**, *24*, 433-440. DOI: 10.1016/0021-9797(67)90241-X.

(3) Cristiano, E.; Hu, Y.-J.; Sigfried, M.; Kaplan, D.; Nitsche, H. A Comparison of Point of Zero Charge Measurement Methodology. *Clays Clay Miner.* **2011**, *59*, 107-115. DOI: 10.1346/CCMN.2011.0590201.

(4) Jang, H.; Lee, W.; Lee, J. Nanoparticle dispersion with surface-modified silica nanoparticles and its effect on the wettability alteration of carbonate rocks. *Colloids Surf. A* **2018**, *554*, 261-271. DOI: 10.1016/j.colsurfa.2018.06.045.

(5) Worthen, A. J.; Tran, V.; Cornell, K. A.; Truskett, T. M.; Johnston, K. P. Steric stabilization of nanoparticles with grafted low molecular weight ligands in highly concentrated brines including divalent ions. *Soft Matter* **2016**, *12*, 2025-2039. DOI: 10.1039/C5SM02787J.

(6) Chengara, A.; Nikolov, A. D.; Wasan, D. T.; Trokhymchuk, A.; Henderson, D. Spreading of nanofluids driven by the structural disjoining pressure gradient. *J. Colloid Interface Sci.* **2004**, *280*, 192-201. DOI: 10.1016/j.jcis.2004.07.005.

(7) Wang, J.; Guo, X. Adsorption isotherm models: Classification, physical meaning, application and solving method. *Chemosphere* **2020**, *258*, 127279. DOI: 10.1016/j.chemosphere.2020.127279.
